# International gestational age-specific centiles for umbilical artery Doppler indices: a longitudinal prospective cohort study of the INTERGROWTH-21^st^ Project

**DOI:** 10.1016/j.ajog.2020.01.012

**Published:** 2020-06

**Authors:** Lior Drukker, Eleonora Staines-Urias, José Villar, Fernando C. Barros, Maria Carvalho, Shama Munim, Rose McGready, Francois Nosten, James A. Berkley, Shane A. Norris, Ricardo Uauy, Stephen H. Kennedy, Aris T. Papageorghiou

**Affiliations:** a Nuffield Department of Women’s & Reproductive Health, University of Oxford, Oxford, United Kingdom; b Oxford Maternal & Perinatal Health Institute, Green Templeton College, University of Oxford, Oxford, United Kingdom; c Centre for Tropical Medicine and Global Health, University of Oxford, Oxford, United Kingdom; d Programa de Pós-Graduação em Saúde e Comportamento, Universidade Católica de Pelotas, Pelotas, Brazil; e Programa de Pós-Graduação em Epidemiologia, Universidade Federal de Pelotas, Pelotas, Brazil; f Faculty of Health Sciences, Aga Khan University, Nairobi, Kenya; g Department of Obstetrics and Gynecology, Aga Khan University Hospital, Karachi, Pakistan; h Shoklo Malaria Research Unit, Mahidol-Oxford Tropical Medicine Research Unit, Faculty of Tropical Medicine, Mahidol University, Mae Sot, Thailand; i KEMRI/Wellcome Trust Research Programme, Kilifi, Kenya; j SAMRC Development Pathway for Health Research Unit, Department of Paediatrics and Child Health, University of Witwatersrand, Johannesburg, South Africa; k Department of Nutrition and Public Health Interventions Research, London School of Hygiene and Tropical Medicine, London, United Kingdom; l Division of Paediatrics, Pontifical Universidad de Chile, Santiago, Chile

**Keywords:** antepartum testing, Doppler, fetal growth restriction, fetal well-being, INTERBIO, INTERGROWTH-21^st^ ultrasound, longitudinal study, multinational study, perinatal morbidity, perinatal mortality, placenta, pulsatility index, reference ranges, resistance index, systolic/diastolic ratio, umbilical artery

## Abstract

**Background:**

Reference values for umbilical artery Doppler indices are used clinically to assess fetal well-being. However, many studies that have produced reference charts have important methodologic limitations, and these result in significant heterogeneity of reported reference ranges.

**Objectives:**

To produce international gestational age-specific centiles for umbilical artery Doppler indices based on longitudinal data and the same rigorous methodology used in the original Fetal Growth Longitudinal Study of the INTERGROWTH-21^st^ Project.

**Study Design:**

In Phase II of the INTERGROWTH-21^st^ Project (the INTERBIO-21st Study), we prospectively continued enrolling pregnant women according to the same protocol from 3 of the original populations in Pelotas (Brazil), Nairobi (Kenya), and Oxford (United Kingdom) that had participated in the Fetal Growth Longitudinal Study. Women with a singleton pregnancy were recruited at <14 weeks’ gestation, confirmed by ultrasound measurement of crown–rump length, and then underwent standardized ultrasound every 5±1 weeks until delivery. From 22 weeks of gestation umbilical artery indices (pulsatility index, resistance index, and systolic/diastolic ratio) were measured in a blinded fashion, using identical equipment and a rigorously standardized protocol. Newborn size at birth was assessed using the international INTERGROWTH-21^st^ Standards, and infants had detailed assessment of growth, nutrition, morbidity, and motor development at 1 and 2 years of age. The appropriateness of pooling data from the 3 study sites was assessed using variance component analysis and standardized site differences. Umbilical artery indices were modeled as functions of the gestational age using an exponential, normal distribution with second-degree fractional polynomial smoothing; goodness of fit for the overall models was assessed.

**Results:**

Of the women enrolled at the 3 sites, 1629 were eligible for this study; 431 (27%) met the entry criteria for the construction of normative centiles, similar to the proportion seen in the original fetal growth longitudinal study. They contributed a total of 1243 Doppler measures to the analysis; 74% had 3 measures or more. The healthy low-risk status of the population was confirmed by the low rates of preterm birth (4.9%) and preeclampsia (0.7%). There were no neonatal deaths and satisfactory growth, health, and motor development of the infants at 1 and 2 years of age were documented. Only a very small proportion (2.8%–6.5%) of the variance of Doppler indices was due to between-site differences; in addition, standardized site difference estimates were marginally outside this threshold in only 1 of 27 comparisons, and this supported the decision to pool data from the 3 study sites. All 3 Doppler indices decreased with advancing gestational age. The 3rd, 5th 10th, 50th, 90th, 95th, and 97th centiles according to gestational age for each of the 3 indices are provided, as well as equations to allow calculation of any value as a centile and z scores. The mean pulsatility index according to gestational age = 1.02944 + 77.7456*(gestational age)^–2^ – 0.000004455*gestational age^3^.

**Conclusion:**

We present here international gestational age-specific normative centiles for umbilical artery Doppler indices produced by studying healthy, low-risk pregnant women living in environments with minimal constraints on fetal growth. The centiles complement the existing INTERGROWTH-21^st^ Standards for assessment of fetal well-being.

Click Supplemental Materials and Video under article title in Contents at **ajog.org**

The umbilical artery waveform, obtained using Doppler ultrasonography, reflects the impedance to blood flow in the fetal compartment of the placenta.^1^^,^^2^ The ability to assess the umbilical artery waveform using Doppler was first described in 1977^3^; just a few years later, Trudinger and Cook^4^ first showed that in normally grown fetuses the impedance decreased with advancing gestation, whereas the impedance increased in growth restricted fetuses. The clinical value of measuring the umbilical artery Doppler is now well-established in high-risk pregnancy as one of the few interventions that reduce perinatal mortality but not in low-risk pregnancies.^5^^,^^6^

**AJOG at a Glance**

**Why was this study conducted?**

Many of the studies that have produced reference charts for umbilical artery Doppler indices have methodologic limitations, which explain the large differences in the centiles for these indices.

**Key findings**

We have produced international gestational age-specific umbilical artery Doppler indices centiles based on the rigorous methodology of the INTERGROWTH-21^st^ Project, using a standardized, population based, prospective, longitudinal approach with long-term follow-up of infants.

**What does this add to what is known?**

The use of international gestational age-specific centiles for Doppler indices should improve the management of high-risk pregnancies and standardize research outcomes in observational and interventional studies involving umbilical artery Doppler.

The approach to umbilical artery Doppler acquisition is standardized.^7^ However, our recent systematic review of the studies that have produced umbilical artery Doppler reference charts found considerable methodologic heterogeneity and limitations in study design, statistical analysis, and reporting.^8^ High potential for bias in studies reporting on umbilical artery Doppler was noted, with only 1 study being multicenter; with only 1 study demonstrating comprehensive quality assurance; and only 1 study reporting that sonographers were blinded to the measurement recorded during the examination. Reference ranges varied significantly with important clinical implications on what is considered normal or abnormal, even when restricting the analysis to the highest-scoring studies.^8^ For example, in the 3 studies with the best methodology, the reported 95th centile of the umbilical artery pulsatility index (PI),^9^, ^10^, ^11^ ranged between 1.28 and 1.48 at 32 weeks’ and between 1.03 and 1.40 at 39 weeks’ gestation. This is important because, apart from absent or reversed end-diastolic flow, Doppler indices are used to monitor high-risk pregnancies over time and contribute to the decisions regarding early delivery. It is easy to see that the differences in what is “normal” or “abnormal” between these studies can result in differences in classification of fetal well-being.

Our aim was to address the methodologic limitations identified in our systematic review,^8^ so as to produce international gestational age-specific centiles for umbilical artery Doppler indices for use alongside the INTERGROWTH-21^st^ Standards for fetal growth,^12^ symphysis–fundal height,^13^ gestational weight gain,^14^ early and late pregnancy dating,^15^ newborn size at birth^16^ and body composition,^17^ and postnatal growth of preterm infants.^18^ To that end, we prospectively collected longitudinal data from pregnant women matching the recruitment criteria of the INTERGROWTH-21^st^ standards at both population and individual level, because they met the World Health Organization (WHO) prescriptive criteria for optimal health, nutrition, education, and socioeconomic status.^19^^,^^20^

## Materials and Methods

INTERGROWTH-21^st^ is an international, multicenter, population-based project. Phase I of the INTERGROWTH-21^st^ Project, conducted between 2009 and 2016, consisted of 9 complementary studies designed to describe optimal human growth and development, based conceptually on the WHO prescriptive approach.^21^ The study sites were 8 urban areas worldwide, with no or low levels of major, known, non-microbiological contamination, that were geographically delimited to ensure the study was population-based.^20^

In the Fetal Growth Longitudinal Study (FGLS), one of the components of the INTERGROWTH-21^st^ Project, we enrolled, before 14 weeks’ gestation, a large cohort of healthy, well-nourished women with a naturally conceived singleton pregnancy who met rigorous individual inclusion criteria.^12^ The specific aim was to monitor their babies prospectively until 2 years of age so as to generate international standards.

Doppler measurements were not included in FGLS for pragmatic reasons in the implementation of such a large multicountry project. However, given the lack of robust data supporting the choice of cut-off points for umbilical artery Doppler indices in clinical practice while assessing complicated pregnancies, we specifically included Doppler measurements in Phase II of the INTERGROWTH-21^st^ Project (the INTERBIO-21st Study, Supplementary S1),^22^ with the aim of producing international gestational age-specific centiles to facilitate standardization of the technique and the clinical decision-making in high-risk pregnancies.

Phase II of the INTERGROWTH-21^st^ Project (The INTERBIO-21st Study)^22^ aims to improve the functional classification of the preterm birth and fetal growth restriction syndromes^23^^,^^24^ through a better understanding of how environmental exposures (eg, HIV, malaria), clinical conditions (eg, preeclampsia), and malnutrition influence patterns of human growth from early pregnancy to childhood. Improvements in phenotypic characterization of these complex syndromes at clinical, molecular, and biochemical levels may help in the development of better screening and prevention strategies. The INTERBIO-21st Study prospectively collected information on pregnancy and perinatal outcomes, newborn anthropometric measures, and the child’s growth and development until 2 years of age using the same protocols, standardized tools, and data-collection systems as in the construction of international fetal growth and newborn size standards in Phase I of the INTERGROWTH-21^st^ Project. In addition, a comprehensive set of biological samples was collected. Details on study sites, population characteristics, study design, methodology, and standardization procedures for the collection of longitudinal clinical data and biological samples have been reported elsewhere.^22^^,^^25^^,^^26^

INTERBIO-21st participants were enrolled following the protocols,^22^ data collections system, and standardization procedures, between 2012 and 2015, from 6 geographically diverse populations worldwide, including 3 of the 8 study sites that also took part in FGLS. Those sites were the cities of Pelotas, Brazil (Hospital Miguel Piltcher, Hospital São Francisco de Paula, Santa Casa de Misericórdia de Pelotas, and Hospital Escola da Universidade Federal de Pelotas), Oxford, United Kingdom (John Radcliffe Hospital), and the Parklands suburb of Nairobi, Kenya (The Aga Khan University Hospital).

The selection criteria at the population level in FGLS were as follows: the areas had to be located at an altitude <1600 m with a low risk of fetal and infant growth and developmental disturbances, as well as an absence or low levels of major, known, non-microbiological contamination. Within each area, all institutions classified locally as “private” or “corporation” hospitals and/or serving the middle to upper socioeconomic population were selected, provided that most institutional deliveries from the target population took place there. Women receiving antenatal care had to plan to deliver in these institutions or in a similar hospital located in the same geographical area.

In the INTERBIO-21st Study, we enrolled women from the 3 original FGLS sites (out of 6 included in INTERBIO-21st), irrespective of their risk profile for adverse pregnancy/perinatal outcomes, provided they were at least 18 years old; their pregnancy was conceived naturally; they initiated antenatal care before 14 weeks’ gestation; and their body mass index was less than 35 to avoid difficulties scanning the overweight.

Umbilical artery Doppler indices were measured in all INTERBIO-21st participants. However, only those women who fulfilled the strict FGLS inclusion criteria of optimal health, nutrition, education, and socioeconomic status contributed data to the present analysis. The aim was to produce centiles using data acquired from healthy, low-risk women comparable with those who participated in FGLS; we have previously adopted this concept and produced an FGLS-like population.^19^

The INTERGROWTH-21^st^ Project was approved by the Oxfordshire Research Ethics Committee ‘C’ (reference: 08/H0606/139), the research ethics committees of the individual participating institutions and the corresponding regional health authorities in which the project was implemented. Participants provided written consent to be involved in the study.

### Standard procedures

We enrolled women between 9^+0^ and 13^+6^ weeks’ gestation as determined by ultrasound measurement of crown–rump length.^15^ Following the dating scan, women were scanned every 5±1 weeks until delivery. At each visit, we obtained fetal biometric measures and, from 22^+0^ weeks’ gestation, 3 umbilical artery Doppler indices: PI (systolic velocity-diastolic velocity/mean velocity), resistance index (RI; systolic velocity-diastolic velocity/systolic velocity), and systolic/diastolic ratio (S/D ratio). The end-diastolic flow was recorded as present, absent, or reversed. Detailed documentation on measurement acquisition protocols, the unique standardization procedures, data-collection forms, and electronic data transfer strategies are available at the study website.^25^

The technique for acquiring the Doppler indices was standardized across sites based on the following criteria: (1) sample taken from a free-floating loop of the umbilical cord; (2) fetal quiescence ensured, ie, absence of significant limb/breathing movements; (3) avoidance of venous signal; (4) magnification of the screen with the zoom box so the umbilical artery occupied no less than 50%; (5) sample gate within the center of the vessel; (6) angle correction employed to ensure angle of insonation of less than 30° and confirmed using color Doppler; (7) sweep speed yielded 4–6 consistent waveforms of similar signal; (8) velocity scale of approximately 75% of the peak systolic velocity; (9) image clarity secured by adjustment of pulse repetition frequency and color gain correction; and (10) the average of 3 waveforms used in the analysis.

The acquisition was repeated if the image quality did not satisfactorily meet all 10 criteria. One image was then selected by the sonographer for all 3 measurements: PI, RI, and S/D ratio.^27^ These were performed via auto-tracing of 3 or more consecutive similar waveforms, from the beginning of the systolic to the end of the diastolic signal, selecting the “limited trace” or “automatic trace” options on the ultrasound machine.

Twenty-four experienced sonographers participated in the study (6 in Brazil, 8 in Kenya, and 10 in the United Kingdom); all were locally accredited and underwent uniform standardization. To avoid expected-value bias, the ultrasound machines were modified so that Doppler measures were not visible to the sonographer on the screen; this was also the case for data collection of the fetal biometric measures. Only at the end of a completed scan were the measures revealed. All scans were performed using identical ultrasound machines (Philips HD-9 and Philips Ultrasound, Bothell, WA) with curvilinear abdominal transducers (C5-2, C6-3, V7-3). Ultrasound data were entered locally and submitted electronically to the study database.^25^ Our umbilical artery Doppler measurements quality control methods have previously been published, which include the interobserver variability on a large sample of measurements.^28^

The infants in the INTERBIO-21st Study were seen at 1 and 2 years of age for a detailed assessment of growth, nutrition, morbidity, and motor development. These data were collected by a certified examiner and by interviewing parents. Achievement of milestones (“sitting without support,” “standing with assistance,” “hand-and-knees-crawling,” “walking with assistance,” “standing alone,” and “walking alone”) were considered satisfactory if the time of achievement was within the expected WHO windows (<99th centile child age for each of the expected windows).^29^^,^^30^

### Statistical methods

Sample size and justification for the present study was performed before analyzing the prospectively collected Doppler data. Sample sizes are based on a balance between pragmatic, biological, and statistical considerations. Statistical considerations focused on the precision and accuracy of a single centile, which we have demonstrated a posteriori that was adequate.^12^^,^^31^ Our selection of the final study sample was mostly guided by biological and pragmatic considerations: the desire to use the same study sites that contributed to the Fetal Growth Standards of the INTERGROWTH 21st Project,^12^ providing continuity across the complete set of standards and the need to follow up infants for evaluation of growth and development to 2 years. Overall, 431 fetuses with 1243 repeated scans were available for analysis which mean that it is (to our knowledge) the largest to date to capture umbilical artery Doppler measures longitudinally in a cohort of pregnancies followed from the first trimester of pregnancy up to 2 years of age. Furthermore, longitudinal studies of fetal growth require half the sample size of a cross-sectional study to estimate a given centile with the same precision.^32^ Hence, our cohort of fetuses, contributing 1243 Doppler measures, has the power equivalent to a sample of 2500 measures in a cross-sectional study.

Following the INTERGROWTH-21^st^ Project policy that has been implemented in all our previous publications, we planned to remove from the analyses values that were either implausible within each study site’s distribution or not within 5 standard deviations (SD) of the mean of the overall gestational-age specific values.^12^^,^^16^ This latter criterion was used, rather than more conservative definitions, to minimize the risk of excluding extreme yet valid cases within a very healthy cohort—a scenario that is made worse whenever measures or indices are not normally distributed and skewed.

First, the heterogeneity in umbilical artery Doppler indices within sites was evaluated using variance component analysis to calculate the percentage of variance in each index due to between-site and within-site differences. Only data from women with 3 or more scans were used for this analysis. Separate multilevel mixed-effects models were fitted with random intercepts for the study-site and the woman levels (with women nested within sites) and adjustment for gestational age (treated as a fixed effect), using the restricted maximum likelihood option in the STATA 15 (StataCorp. 2017; StataCorp LLC, College Station, TX) *mixed* module.

Second, similarities between sites were measured using standardized site differences (SSDs), defined as the site mean of each Doppler index minus the pooled mean for all sites relative to the SD of all sites together, adjusted by the gestational age at which the scan was performed within 3 prespecified windows: 23–28, 29–33, and 34–41 weeks’ gestation. In line with previous publications,^22^^,^^33^ pooling the data from different sites was considered appropriate if differences were less than 0.5 SD of the pooled means for each gestational age and measure.

Different distributions and smoothing techniques were explored for the construction of the curves using the GAMLSS (Generalized Additive Models for Location, Scale and Shape) package in R^34^ and the *xriml* module in STATA.^35^ Starting with the simplest model assuming a normal distribution, goodness of fit was evaluated using the Akaike information criteria,^36^ quantile-quantile (q-q) plot of residuals, plots of residuals vs fitted values, and the distribution of fitted z scores across gestational ages to decide if modelling complexity needed to be increased.

In summary, the exponential normal distribution^35^ with second-degree fractional polynomial smoothing^37^ was as good as more complex methods with a greater number of parameters that account for skewness and kurtosis in the distribution of the values.^38^ Models fitted in a multilevel framework accounting for repeated measurements showed little impact on the estimated centiles. Goodness of fit for the overall models was assessed by comparing empirical centiles (calculated per completed gestational week) with fitted centiles.

Eleven scans, performed on 10 women before 23 weeks’ gestation, were excluded from the analysis to avoid edge effects contributing to undesirable model fit at lower gestational ages. Twenty-two scans performed at 23 and 41 weeks’ gestation were included in the modeling to stabilize the curves at the tails of the gestational age range. However, reporting was restricted to the period between 24 and 40 weeks’ gestation, which represents the window of established clinical utility.

## Results

### Population

**Figure 1:**
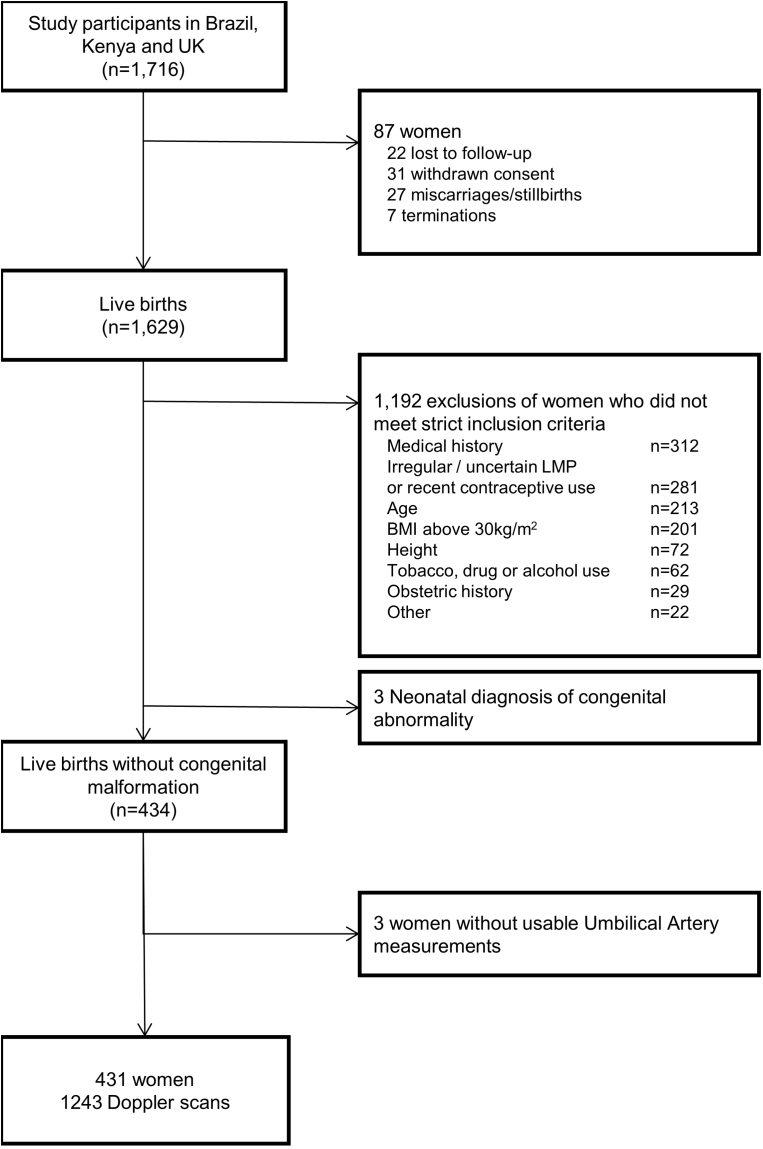
Flow chart of participants in the study *Drukker et al. International gestational age-specific centiles for umbilical artery Doppler indices: a longitudinal prospective cohort study of the INTERGROWTH-21^st^ Project. Am J Obstet Gynecol 2020.*

Among the 1716 women enrolled at the three INTERBIO-21st Study sites who also participated in FGLS, 87 were excluded because of loss to follow-up, withdrawn consent, termination or pregnancy loss, leaving 1629 with live singleton births. Of these, 434 (27%) fulfilled the FGLS individual criteria, which is similar to the proportion seen in the original fetal growth longitudinal study.^12^ Three women had babies with a postnatal diagnosis of a congenital abnormality and also were excluded, resulting in data for analysis from 431 women who had 1243 ultrasound scans (Figure 1). The contribution of each site to the total study population was 88 women from Brazil (20.4%), 219 from Kenya (50.8%), and 124 from the United Kingdom (28.8%).

From 24 to 40 weeks’ gestation, there were between 20 and 119 individual scans per gestational week. The median number of umbilical artery Doppler scans per woman was 3 (range 1–5), with 319 women (74.0%) having 3 or more measurements. As planned, based on the INTERGROWTH-21^st^ policy, we excluded 6 measures because they were not within 5 SD of the mean of the overall gestational-age specific values. Removing this small number of outliers had no effect on the centiles.

The maternal, pregnancy and newborn characteristics of the women who contributed data to the present analysis (Table 1) were strikingly similar to the baseline characteristics of the original FGLS population whose data were used to produce the international INTERGROWTH-21^st^ Fetal Growth Standards.^12^

**Table 1:** Maternal, pregnancy, and newborn characteristics of the study population

	FGLS n=4321	Present study n=431
Maternal age, y	28.4 (3.9)	28.9 (3.7)
Maternal height, cm	162.2 (5.8)	163.9 (5.7)
Maternal weight, kg	61.3 (9.1)	64.3 (8.7)
Paternal height, cm	174.4 (7.3)	176.8 (6.8)
Body mass index, kg/m^2^	23.3 (3.0)	23.9 (2.9)
Gestational age at first visit, wk	11.8 (1.4)	12.0 (1.1)
Years of formal education, y	15.0 (2.8)	15.4 (2.7)
Hemoglobin level at <15 wk, g/L	125 (11)	128.8 (9.3)
Married or cohabiting	4204 (97%)	400 (93%)
Nulliparous	2955 (68%)	259 (60%)
Preeclampsia	31 (<1%)	3 (<1%)
Pyelonephritis	16 (<1%)	3 (<1%)
Any sexually transmitted infection	3 (<1%)	17 (4%)
Spontaneous initiation of labor	2868 (66%)	266 (62%)
Preterm premature rupture of membranes (<37 wk)	80 (2%)	9 (2%)
Cesarean delivery	1541 (36%)	159 (37%)
Neonatal intensive care unit admission >1 d	240 (6%)	24 (6%)
Preterm (<37 wk gestation)	195 (5%)	21 (5%)
Preterm and spontaneous onset of labor	126 (3%)	10 (2%)
Term low birth weight (<2500 g; ≥37 wk gestation)	128 (3%)	11 (3%)
Neonatal mortality	7 (<1%)	0 (0%)
Male sex	2149 (50%)	232 (54%)
Exclusive breastfeeding at discharge	3786 (88%)	399 (93%)
Mother admitted to intensive care unit	17 (<1%)	3 (<1%)
Newborn weight (≥37 wk gestation), kg	3.3 (0.4)	3.3 (0.5)
Newborn length (≥37 wk gestation), cm	49.4 (1.9)	49.4 (1.9)
Newborn head circumference (≥37 wk gestation), cm	33.9 (1.3)	34.5 (1.2)

Data are mean (standard deviation) or number (percent).

*FGLS*, Fetal Growth Longitudinal Study of the INTERGROWTH 21st Project.

*Drukker et al. International gestational age-specific centiles for umbilical artery Doppler indices: a longitudinal prospective cohort study of the INTERGROWTH-21^st^ Project. Am J Obstet Gynecol 2020*.

**Figure 2:**
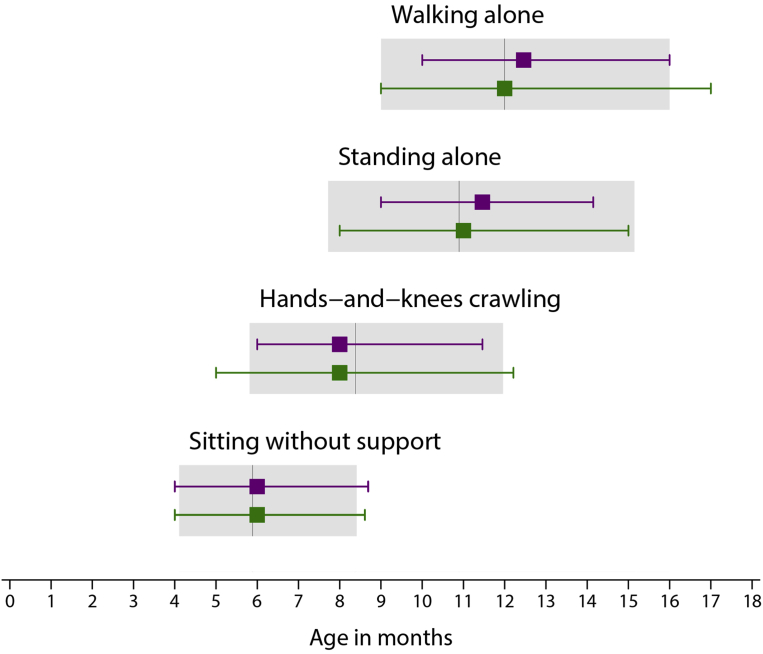
Gross motor development milestones for included infants Median age of achievement (3rd and 97th centiles) of 4 gross motor development milestones for infants that were included in the INTERGROWTH-21^st^ Fetal Growth Standards (*purple*) and those included in the present analysis (*green*). For comparison, the median, 3rd and 97th centiles of the WHO windows of achievement for the same milestones are presented as *gray bars*. *Drukker et al. International gestational age-specific centiles for umbilical artery Doppler indices: a longitudinal prospective cohort study of the INTERGROWTH-21^st^ Project. Am J Obstet Gynecol 2020.*

Assessment of the infants at 1 (n=329; 76%) and 2 years of age (n=319; 74%) confirmed their adequate health and nutritional status (Table 2), and that their developmental milestones were reached at a similar age to the infants in the original FGLS, all within the WHO-recommended range for these gross motor milestones.^29^^,^^30^ (Figure 2).

**Table 2:** Morbidity in the second year of life for the 319 infants in the analysis

Morbidity in the second year of life	Infants in the analysis (n=319)^a^
Hospitalized at least once	27 (8.5)
Any prescription made by a health care practitioner	275 (86.2)
Antibiotics (≥3 regimens)	55 (17.2)
Iron/folic acid/vitamin B12/other vitamins (≥3 regimens)	72 (22.6)
Up-to-date with local vaccination policies	313 (98.1)
Otitis media/pneumonia/bronchiolitis	42 (13.2)
Parasitosis/diarrhea/vomiting	15 (4.7)
Exanthema/skin disease	88 (27.6)
Urinary tract infection/pyelonephritis	2 (0.6)
Fever ≥3 days (≥3 episodes)	36 (11.3)
Other infections requiring antibiotics	5 (1.6)
Asthma	11 (3.4)
Gastroesophageal reflux	6 (1.9)
Cow’s milk protein allergy	6 (1.9)
Food allergies	9 (2.8)
Injury trauma	22 (6.9)
Surgery	8 (2.5)

Data are number (%). Missing data below 1% for all variables.

*Drukker et al. International gestational age-specific centiles for umbilical artery Doppler indices: a longitudinal prospective cohort study of the INTERGROWTH-21^st^ Project. Am J Obstet Gynecol 2020.*

a For 5 infants, information on morbidities in the first year of life was used.

### Doppler indices

**Figure 3:**
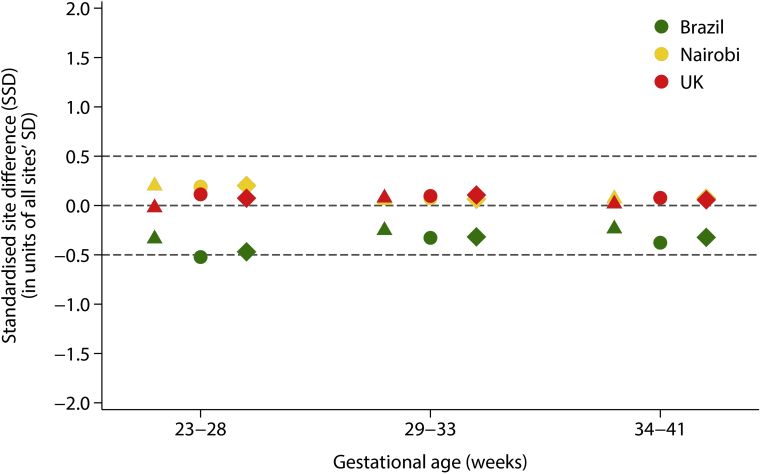
Standardized site differences (SSD) for three umbilical artery Doppler indices SSDs for 3 umbilical artery Doppler indices for pulsatility index (*triangles*), resistance index (*circles*), and for the systolic/diastolic ratio (*diamonds*). SSDs were calculated as the site mean of each index minus the pooled mean divided by the standard deviation of all sites together, adjusted at the mean gestational age for the specified window. *SSD*, standardized site difference. *Drukker et al. International gestational age-specific centiles for umbilical artery Doppler indices: a longitudinal prospective cohort study of the INTERGROWTH-21^st^ Project. Am J Obstet Gynecol 2020.*

For the 3 Doppler indices, the percentage of the total variance due to the difference between study sites was 2.8%–6.5%, whereas the percentage of total variance explained by differences between individuals within a site ranged from 20.9% to 25.4%. In other words, the within-site percentage variance was 4–6 times greater than the between site percentage of the total variance (Supplemental Table 1). From the 27 comparisons made across gestational age, only 1 SSD estimate was marginally outside this threshold: RI SSD for Brazil in the 23-28 weeks’ gestational age window = –0.52, (Figure 3). These 2 findings strongly supported the decision to pool the data from the three study sites to produce the international gestational age-specific normative centiles.

**Figure 4:**
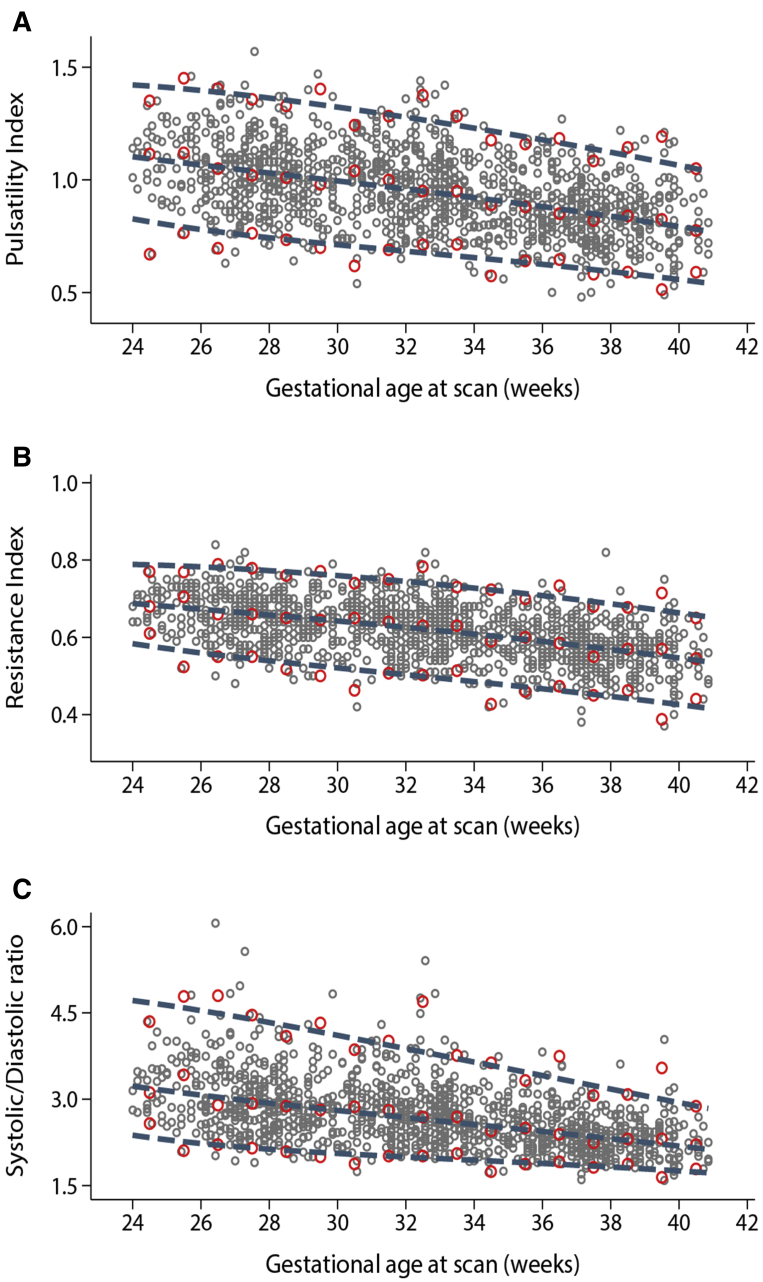
Smoothed 3rd, 50th, and 97th centile curves of umbilical artery Doppler indices Fitted centiles according to gestational age in weeks are presented as *blue dashed lines* for (**A**) pulsatility index, (**B**) resistance index, and (**C**) systolic/diastolic ratio. *Red circles* show empirical 3rd, 50^th^, and 97th centiles for each completed week of gestation; *gray circles* show individual observations. *Drukker et al. International gestational age-specific centiles for umbilical artery Doppler indices: a longitudinal prospective cohort study of the INTERGROWTH-21^st^ Project. Am J Obstet Gynecol 2020.*

The gestational-age specific 3rd, 50th, and 97th fitted centiles for each of the Doppler indices are shown in Figure 4, along with the observed centiles for each completed week. The comparison between smoothed and empirical centiles suggests that the models have reasonable fit to the data. Gestational age-specific standard values for use in clinical practice for the 3rd, 5th, 10th, 50th, 90th, 95th, and 97th centiles of umbilical artery PI, RI, and S/D ratio are presented in Table 3, Table 4, Table 5. The corresponding regression equations for the model parameters are presented in Table 6, along with the equations to calculate z scores and centiles.

**Table 3:** Umbilical artery pulsatility index (PI) centile values according to gestational age

Gestational age (weeks + days)	Centile						
	3rd	5th	10th	50th	90th	95th	97th
24+0	0.83	0.86	0.91	1.10	1.31	1.38	1.42
25+0	0.80	0.84	0.89	1.08	1.30	1.37	1.41
26+0	0.78	0.81	0.87	1.07	1.29	1.35	1.40
27+0	0.76	0.79	0.85	1.05	1.27	1.34	1.38
28+0	0.74	0.78	0.83	1.03	1.25	1.32	1.36
29+0	0.73	0.76	0.81	1.01	1.23	1.30	1.34
30+0	0.71	0.75	0.80	1.00	1.21	1.28	1.32
31+0	0.70	0.73	0.78	0.98	1.19	1.26	1.30
32+0	0.68	0.72	0.77	0.96	1.17	1.24	1.28
33+0	0.67	0.70	0.75	0.94	1.15	1.21	1.25
34+0	0.66	0.69	0.74	0.92	1.13	1.19	1.23
35+0	0.64	0.67	0.72	0.90	1.10	1.16	1.20
36+0	0.63	0.66	0.70	0.88	1.08	1.14	1.18
37+0	0.61	0.64	0.69	0.86	1.05	1.11	1.15
38+0	0.59	0.62	0.67	0.84	1.03	1.08	1.12
39+0	0.58	0.60	0.65	0.82	1.00	1.06	1.09
40+0	0.56	0.59	0.63	0.79	0.97	1.03	1.06

*Drukker et al. International gestational age-specific centiles for umbilical artery Doppler indices: a longitudinal prospective cohort study of the INTERGROWTH-21^st^ Project. Am J Obstet Gynecol 2020*.

**Table 4:** Umbilical artery resistance index centile (RI) values according to gestational age

Gestational age (weeks + days)	Centile						
	3rd	5th	10th	50th	90th	95th	97th
24+0	0.58	0.60	0.62	0.69	0.76	0.78	0.79
25+0	0.57	0.58	0.61	0.68	0.75	0.77	0.79
26+0	0.56	0.57	0.60	0.67	0.75	0.77	0.78
27+0	0.55	0.56	0.59	0.67	0.74	0.76	0.78
28+0	0.54	0.55	0.58	0.66	0.74	0.76	0.77
29+0	0.53	0.55	0.57	0.65	0.73	0.75	0.77
30+0	0.52	0.54	0.56	0.64	0.72	0.75	0.76
31+0	0.51	0.53	0.55	0.63	0.72	0.74	0.75
32+0	0.50	0.52	0.54	0.63	0.71	0.73	0.74
33+0	0.49	0.51	0.53	0.62	0.70	0.72	0.74
34+0	0.49	0.50	0.52	0.61	0.69	0.71	0.73
35+0	0.48	0.49	0.52	0.60	0.68	0.70	0.72
36+0	0.47	0.48	0.51	0.59	0.67	0.69	0.71
37+0	0.46	0.47	0.50	0.58	0.66	0.68	0.70
38+0	0.45	0.46	0.49	0.57	0.65	0.67	0.69
39+0	0.44	0.45	0.48	0.56	0.64	0.66	0.68
40+0	0.43	0.44	0.46	0.55	0.63	0.65	0.66

*Drukker et al. International gestational age-specific centiles for umbilical artery Doppler indices: a longitudinal prospective cohort study of the INTERGROWTH-21^st^ Project. Am J Obstet Gynecol 2020*.

**Table 5:** Umbilical artery systolic/diastolic ratio (S/D Ratio) centile values according to gestational age

Gestational age (weeks + days)	Centile						
	3rd	5th	10th	50th	90th	95th	97th
24+0	2.38	2.46	2.61	3.23	4.12	4.46	4.72
25+0	2.30	2.39	2.53	3.15	4.03	4.38	4.63
26+0	2.23	2.32	2.46	3.07	3.95	4.29	4.54
27+0	2.18	2.26	2.40	3.00	3.86	4.19	4.44
28+0	2.13	2.22	2.35	2.93	3.77	4.09	4.33
29+0	2.09	2.17	2.31	2.87	3.68	3.99	4.22
30+0	2.06	2.14	2.26	2.81	3.58	3.89	4.11
31+0	2.03	2.10	2.22	2.74	3.49	3.78	4.00
32+0	2.00	2.07	2.19	2.68	3.40	3.67	3.88
33+0	1.97	2.04	2.15	2.62	3.30	3.57	3.76
34+0	1.94	2.01	2.11	2.56	3.21	3.46	3.65
35+0	1.91	1.97	2.08	2.50	3.12	3.35	3.53
36+0	1.88	1.94	2.04	2.44	3.02	3.24	3.41
37+0	1.85	1.91	2.00	2.38	2.93	3.14	3.30
38+0	1.82	1.87	1.96	2.32	2.83	3.03	3.18
39+0	1.79	1.84	1.92	2.25	2.73	2.92	3.06
40+0	1.75	1.80	1.87	2.19	2.64	2.81	2.94

*Drukker et al. International gestational age-specific centiles for umbilical artery Doppler indices: a longitudinal prospective cohort study of the INTERGROWTH-21^st^ Project. Am J Obstet Gynecol 2020*.

**Table 6:** Equations for parameters and computations of z scores and centiles for 3 umbilical artery Doppler indices according to GA, in weeks

Parameter	Equation
Skewness	Pulsatility index λ(GA) = –0.0768617 Resistance index λ(GA) = 0.0172944 Systolic/diastolic ratio λ(GA) = –0.2752483
Mean	Pulsatility index μ(GA) = 1.02944 + 77.7456*GA^–2^ – 0.000004455*GA^3^ Resistance index μ(GA) = 0.674914 + 25.3909*GA^–2^ – 0.0000022523*GA^3^ Systolic/diastolic ratio μ(GA) = 2.60358 + 445.991*GA^–2^ – 0.0000108754*GA^3^
Coefficient of variation	Pulsatility index σ(GA) = –0.00645693 + 254.885*ln(GA)*GA^–2^ – 715.949*GA^–2^ Resistance index σ(GA) = 0.0375921 + 60.7614*ln(GA)*GA^–2^ – 183.336*GA^–2^ Systolic/diastolic ratio σ(GA) = –0.503202 + 1268.37*ln(GA)*GA^–2^ – 3417.37*GA^–2^
z score	z = λ^–1^∗{exp[(y-µ)∗ λ∗ σ^–1^]–1}
Centile	c = normal(z) * 100

*GA*, Gestational age in exact weeks; *ln*, natural logarithm; *y*, Doppler index value.

Example: calculating the pulsatility index centile at a certain GA

Measurement: pulsatility index = 1.00 at GA 36+4 Calculations: y = 1.00 36+4 = 256 days GA = 256 / 7 = 36.571429 (exact weeks) λ = –0.0768617 μ = 1.02944 + 77.7456*(36.571429)^–2^ – 0.000004455*(36.571429)^3^ = 1.02944 + 77.7456*0.00074768 – 0.000004455*48913.168 = 1.02944 + 0.05812883 – 0.21790816 = 0.869660 σ = –0.00645693 + 254.885*ln(36.571429)* (36.571429)^–2^ – 715.949*(36.571429)^–2^ = –0.00645693 + 254.885*3.5992673*0.00074768 – 715.949*0.00074768 = 0.144163 Z = λ^–1^*{exp[(y-μ)* λ* σ^–1^] – 1} = (–0.0768617)^–1^*{exp[(1.00-0.86966067)* –0.0768617*(0.14416339)^–1^] – 1} = –13.010381*{exp[0.13033933*–0.0768617*6.9365738] – 1} = –13.010381*{exp[-0.06949131] – 1} = –13.010381*{0.93286824– 1} = 0.87340977 c = normal(0.87340977)* 100 = 80.9 Conclusion: A pulsatility index value of 1.00 measured at 36+4 gestational weeks has a z score of 0.87 and is placed at the 80.9th centile of the distribution.

*Drukker et al. International gestational age-specific centiles for umbilical artery Doppler indices: a longitudinal prospective cohort study of the INTERGROWTH-21^st^ Project. Am J Obstet Gynecol 2020*.

## Discussion

### Principal findings

We have presented here a set of normative values for the interpretation of Doppler measures in the clinical care of high-risk pregnancies. These are based on serial ultrasound measures, obtained prospectively from low-risk, singleton pregnancies in healthy women from 3 geographically delimited, diverse populations. They match, in their study population and methodology, the comprehensive set of tools previously published for the standardized assessment of fetal, pregnancy, newborn, infant, and child growth and developmental parameters. In the present analysis, we have overcome the methodologic limitations of previous studies by meeting 22 of the 24 criteria used to evaluate their quality in our systematic review.^8^ Crucially, the data were collected from 3 diverse populations in the context of a large-scale project to standardize fetal, neonatal, and infant monitoring tools, whereas, with one exception,^11^ all past studies were performed in a single hospital with limited relation to other pregnancy parameters or newborn and infant follow up. Remarkably, the proportion of low-risk pregnancies (around 30%), the low adverse outcome rates including preterm birth, and results of long-term follow-up were similar to the previously observed samples of the INTERGROWTH 21st Project, demonstrating the interoperability of these basic biological makers when health, nutrition, and socioeconomic conditions are adequate.

### Results

We have confirmed that Doppler indices fall with advancing gestational age as the physiological adaptation of the umbilical–placental bed leads to a decrease in vascular flow resistance.^39^ A failure in this physiological process results in increased vascular resistance, evidenced by a fall in diastolic flow. In combination with the increasing demands of the growing fetus on the placenta, there is an increase in PI, RI, and S/D ratios.

Uniquely in the literature, we were interested to document that the studied fetuses were clinically healthy at birth and up to 2 years to support the concept that they were eligible for the construction of normative values. We explore this question by assessing the health, growth, and development of the infants up until 2 years of age, as has been the policy with all our standards.^18^ We strongly believe that the failure to follow up infants enrolled in perinatal studies in general and in ultrasound studies specifically, particularly those focused on fetal well-being, has been a major shortcoming of our specialty. The finding of satisfactory growth, health, and neurodevelopmental outcomes at 2 years of age, evaluated by researchers masked of the hypotheses tested in the present study, that we have prospectively documented, should provide clinicians with confidence regarding: (1) the appropriateness of selecting our study population for determining normative values, and (2) the use of the presented centiles in clinical practice during fetal well-being assessment, validated against outcomes of long-term relevance.

### Clinical implications

In summary, we propose 2 take-home messages: first, from a biological perspective, we have shown that the fetoplacental circulation functions, expressed by these Doppler indexes, are similar across different populations when optimal health, uncomplicated pregnancies, nutritional, and environmental conditions are met. As previously reported for early and late fetal, newborn, preterm postnatal growth, infant, and child skeletal growth, maternal weight gain, symphysis fundal growth, cerebellum and Sylvian fissure maturation, neurodevelopment and related behaviors, and by WHO for term infants and children, the proportional magnitude of the variance in the Doppler indices between fetal cohorts from these different study sites is very small (around 5% of the total variance) as compared with the large proportion of the total variance explained between fetuses within a study site. This evidence confirms the similarities in fundamental biological human characteristics across regions, ethnic groups, and ancestries.

Second, the current reference charts for clinical interpretation of umbilical Doppler indices demonstrate large differences in the 95th centile values, which may be having an adverse effect on perinatal outcomes. It is certainly very difficult to generate high-quality, evidence-based guidelines for the management of the compromised fetus and coordinate referral systems when an important component of the clinical armamentarium offers normal PI value in one chart that is above 2 SDs on another.^40^ These inconsistencies should concern clinicians and parents alike. The lack of standardization, which pervades obstetric practice, is probably not found in any other field of medicine that involves such important decision-making. A strong commitment is required in our profession to avoid retaining these patterns of care.

### Research implications

In the current literature, there are large differences in umbilical artery cut-offs. This has several implications for research: individual studies where an abnormal umbilical artery Doppler index is used as an enrolment criterion may be difficult to combine depending on what reference is used, whereas in multicenter studies, charts used in different institutions may lead to heterogeneous participant selection. The same is true of course where a Doppler index is used as a diagnostic criterion (umbilical PI >95th centile figures as a criterion for both early and late growth restriction), or to guide an intervention, such as delivery; a recent study has shown that differences in umbilical artery PI cut-off values would result in differential management in a cohort of small-for gestational age fetuses from 20% to 40%.^40^

This need to standardize practice is not only relevant to clinical management but also research into fetal growth restriction as we have proposed before.^41^^,^^42^ We now need to start defining not just what to measure, but how to measure it; in time, this will allow harmonization of care, research, and aid better data synthesis of evidence in future.

### Strengths and limitations

Our work has several unique features and strengths. First, the ultrasound data were obtained with the same degree of scientific rigor, standardization, and quality assurance as in the fetal growth during pregnancy standards we have published,^43^, ^44^, ^45^ including using identical ultrasound equipment at each site and a single validated acquisition protocol. Uniquely, we have masked all Doppler values to the sonographers, reducing “expected values” bias often recognized in this field.^45^, ^46^, ^47^

Second, our achieved sample size compared favorably with the published literature^8^: our study involved 431 fetuses with 1243 repeated scans which mean it is, to our knowledge, the largest to date to capture umbilical artery Doppler measures longitudinally in a cohort of pregnancies followed from the first trimester of pregnancy up to 2 years of age. Furthermore, longitudinal studies of fetal growth require half the sample size of a cross-sectional study to estimate a given centile with the same precision.^32^ Hence, our cohort of fetuses, contributing 1243 Doppler measures, has the power equivalent to a sample of 2500 measures in a cross-sectional study. This is reflected in the high level of precision we achieved in the estimation of the centiles, ie, the width of the 95% confidence intervals, when compared with the range of expected values at that gestational age. For example, at the clinically relevant gestational age of 34^+0^ weeks, when decisions are made partially based on Doppler values, for the 50th centile the width of the 95% confidence interval was 0.02, 0.01, and 0.07 for PI, RI, and S/D ratio, respectively. The values for the 95th and 97th centiles were 0.04, 0.01, and 0.16 and 0.04, 0.02, and 0.22, respectively.

We accept that the work has limitations. There were 2 of the 24 criteria that we identified in our systematic review as required for the construction of ultrasound normative charts, with which we did not comply: the first is that each Doppler measure was only taken once despite our recommendation that ultrasound measures for the construction of standards should be taken in triplicate and the average used in the analyses,^8^ as we have done for all previous standards.^12^, ^13^, ^14^, ^15^, ^16^, ^17^, ^18^ We took only single Doppler measures because, although Doppler ultrasound is considered safe, we felt it was important, in the absence of any obstetric indication, to minimize fetal insonation in a research study, based on the as low as reasonably achievable principle.^48^

The second limitation was that we did not perform an inter- and intraobserver evaluation. Nevertheless, we have undertaken strict quality assessment using a scoring system that was used in this study and this has been shown to be more reproducible than subjective assessment^28^; therefore, all possible measures to improve reproducibility have been addressed.

We did not examine other Doppler parameters that are suggested in maternal–fetal medicine and concentrate on those that are widely used. This was done mainly because this was an already-complex prospective study and it is recognized that the addition of more measures and examinations to healthy subjects burdens participants and reduces follow-up compliance; it also increases observers’ measure error. It has been our policy to concentrate on the most used perinatal practices as priority for standardization of clinical practice across medical specialties. We hope that our work should encourage other researchers to adopt a similar approach to other parameters such as the cerebroplacental flow ratio. There is promising evidence that such evaluation could help to predict adverse perinatal and/or neurodevelopmental outcomes in growth-restricted fetuses.^49^

Comparisons with presently used charts are a challenge because of the methodologic limitations,^8^ including lack of standardization of equipment and measurement methods, pregnancy outcomes, the unreliability of gestational age estimates, observer bias, and limited information of the underlying population served by mostly high-risk hospitals. Perhaps, as a result of these limitations, some of the observed patterns are not plausible, for example, large ups and downs in the values according to gestational age.^11^^,^^50^ In addition, INTERGROWTH-21^st^ centiles are not intended for comparison with all single hospital charts produced because that will be a never ending process considering the number of institutions around the world producing such local charts. The task is to create normative values from prescriptive populations that are compatible with adequate fetal growth, pregnancy, and neonatal outcomes and that are associated with adequate child growth, health and development.

## Conclusion

In conclusion, to overcome the limitations of previous ultrasound studies and standardize clinical practice, we adopted a prescriptive approach to the production of international gestational age-specific centiles for umbilical artery Doppler indices. The work has contributed a helpful clinical tool for the assessment of fetal well-being and placental function in high-risk pregnancies, which complements the existing INTERGROWTH-21^st^ tools for monitoring growth and development from early pregnancy to 2 years of age.^12^, ^13^, ^14^, ^15^, ^16^, ^17^, ^18^ The healthy outcomes we report at 2 years of age in the infants whose intrauterine growth and Doppler indices we so rigorously evaluated should give clinicians and parents confidence in the benefits of using the centiles in clinical practice to manage high-risk pregnancies.

## Figures and Tables

**Supplemental Table 1:** Variance components analysis^a^ for 3 umbilical artery Doppler indices

	Pulsatility index	Resistance index	Systolic/diastolic ratio
Number of measures	1021	1020	1022
Variance between study sites	2.8	6.5	5.6
Variance between individuals within a site	25.4	22.9	20.9
Residual variance	71.8	70.6	73.5

Models included women with 3 or more scans performed between 23 and 40 weeks’ gestation (316 women and 1023 scans were available for these analyses).

*Drukker et al. International gestational age-specific centiles for umbilical artery Doppler indices: a longitudinal prospective cohort study of the INTERGROWTH-21^st^ Project. Am J Obstet Gynecol 2020*.

a From multilevel mixed-effects models fitted with random intercepts for the study-site and individual levels (with individuals nested within sites) and adjustment for gestational age (treated as fixed effect).

## Supplementary Data

Umbilical artery Doppler

Umbilical artery Doppler

## Members of the INTERBIO-21st Committees, participating countries and local investigators

**Scientific Advisory Committee**

M. Katz (Chair), M. K. Bhan, C. Garza, A. Langer, P. M. Rothwell, S. Zaidi

**Steering Committee**

R. Uauy (Chair), S. Kennedy (Co-Principal Investigator), J. Villar (Co-Principal Investigator), D. G. Altman, F. C. Barros, J. Berkley, F. Burton, M. Carvalho, L. Cheikh Ismail, W. C. Chumlea, A. Lambert, S. Munim, S. Norris, F. Nosten, A. T. Papageorghiou, C. Victora

**Executive Committee**

J. Villar (Chair), D. G. Altman, L. Cheikh Ismail, R. Craik, S. Kennedy, A. Lambert, J. A. Noble, A. T. Papageorghiou, R. Uauy

**Study Coordinating Unit**

J. Villar (Head), S. Ash, R. Craik, L. Cheikh Ismail, S. Kennedy, A. Lambert, A. T. Papageorghiou, M. Shorten

**Data Analysis Group**

D. G. Altman (Head), S. Kennedy, E. O. Ohuma, E. Staines Urias, J. Villar

**Data Management Group**

D. G. Altman (Head), I. Ahmed, S. Ash, C. Condon, M. Mainwaring, D. Muninzwa, M. F. da Silveira, E. Staines Urias, L. Walusuna, S. Wiladphaingern

**Ultrasound Group**

A. T. Papageorghiou (Head), L. Salomon (Senior external advisor), M. Buckle, N. Jackson, A. Mitidieri, S. Munim, H. Mwangudzah, R. Napolitano, T. Norris, J. Sande, J. Shah, G. Zainab

**Anthropometry Group**

L. Cheikh Ismail (Head), W. C. Chumlea (Senior external advisor), J. Kizidio, B. Monyepote, F. Puglia, M. Salim, R. Salam, V. I. Carrara

**Laboratory Group**

R. Craik (Head), D. Alam, Y. Guman, J. Kilonzo, A. Min, V. Ngami, I. Olivera, G. Deutsch

**Neonatal Group**

Z. A. Bhutta (Head), E. Bertino, F. Giuliani, R. Uauy

**Environmental Health Group**

B. Eskenazi (Head), J. Villar

**Neurodevelopment Group**

A. Stein (Head), M. Fernandes (Coordinator), A. Abubakar, J. Acedo, L. Aranzeta, L. Cheikh Ismail, F. Giuliani, D. Ibanez, S. Kennedy, M. Kihara, E. de Leon, C. R. Newton, S. Savini, A. Soria- Frisch, J. Villar, K. Wulff

**INTERBIO-21^st^ participating countries and local investigators**

*Brazil*: F. C. Barros (Principal Investigator), M. Domingues, S. Fonseca, A. Leston, A. Mitidieri, D. Mota, I. K. Sclowitz, M. F. da Silveira

*Kenya (Kilifi)*: J. Berkley (Principal Investigator), B. Kemp, H. Barsosio, S. Mwakio, H. Mwangudzah, V. Ngami, M. Salim, A. Seale, L. Walusuna

*Kenya (Nairobi)*: M. Carvalho and W. Stones (Co-Principal Investigators), D. Muninzwa, J. Kilonzo, J. Kizidio, R. Ochieng, J. Sande, J. Shah

*Pakistan*: S. Munim and G. Zainab (Co-Principal Investigators), I. Ahmed, D. Alam, A. Raza, R. Salam

*South Africa*: S. Norris (Principal Investigator), Y. Guman, T. Lephoto, S. Macauley, L. Malgas

*Thailand*: F. Nosten (Principal Investigator), N. Jackson, R. McGready, A. Min, V. I. Cararra, S. Wiladphaingern

*United Kingdom*: S. Kennedy (Principal Investigator), S. Ash, M. Baricco, A. Capp, L. Cheikh Ismail, R. Craik, S. Hussein, A. Laister, A. Lambert, T. Lewis, E. Maggiora, R. Napolitano, T. Norris, A. T. Papageorghiou, B. Patel, F. Puglia, F. Roseman, S. Roseman, M. Sharps, A. Varalda, R. Carew
