# Supplementary material for: International gestational age-specific centiles for umbilical artery Doppler indices: a longitudinal prospective cohort study of the INTERGROWTH-21st Project
Source: Am J Obstet Gynecol. 2020 Jun;222(6):602.e1–602.e15. doi: 10.1016/j.ajog.2020.01.012 (PMC7287403; doi:10.1016/j.ajog.2020.01.012)
Supplement: Umbilical artery Doppler [file mmc2.pptx]

## Slide 1
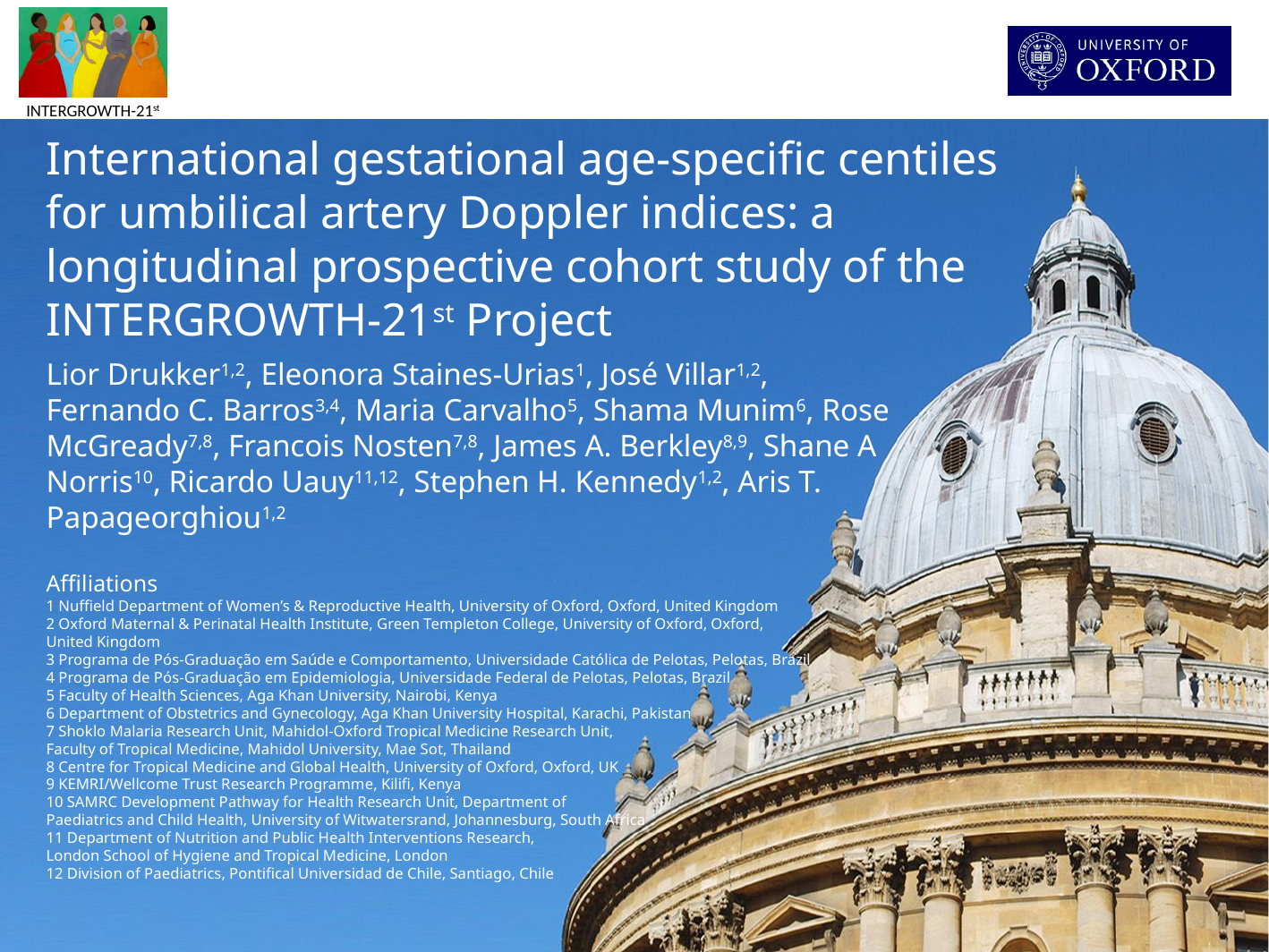

International gestational age-specific centiles for umbilical artery Doppler indices: a longitudinal prospective cohort study of the INTERGROWTH-21st Project
Lior Drukker1,2, Eleonora Staines-Urias1, José Villar1,2, Fernando C. Barros3,4, Maria Carvalho5, Shama Munim6, Rose McGready7,8, Francois Nosten7,8, James A. Berkley8,9, Shane A Norris10, Ricardo Uauy11,12, Stephen H. Kennedy1,2, Aris T. Papageorghiou1,2
Affiliations
1 Nuffield Department of Women’s & Reproductive Health, University of Oxford, Oxford, United Kingdom
2 Oxford Maternal & Perinatal Health Institute, Green Templeton College, University of Oxford, Oxford,
United Kingdom
3 Programa de Pós-Graduação em Saúde e Comportamento, Universidade Católica de Pelotas, Pelotas, Brazil
4 Programa de Pós-Graduação em Epidemiologia, Universidade Federal de Pelotas, Pelotas, Brazil
5 Faculty of Health Sciences, Aga Khan University, Nairobi, Kenya
6 Department of Obstetrics and Gynecology, Aga Khan University Hospital, Karachi, Pakistan
7 Shoklo Malaria Research Unit, Mahidol-Oxford Tropical Medicine Research Unit,
Faculty of Tropical Medicine, Mahidol University, Mae Sot, Thailand
8 Centre for Tropical Medicine and Global Health, University of Oxford, Oxford, UK
9 KEMRI/Wellcome Trust Research Programme, Kilifi, Kenya
10 SAMRC Development Pathway for Health Research Unit, Department of
Paediatrics and Child Health, University of Witwatersrand, Johannesburg, South Africa
11 Department of Nutrition and Public Health Interventions Research,
London School of Hygiene and Tropical Medicine, London
12 Division of Paediatrics, Pontifical Universidad de Chile, Santiago, Chile

## Slide 2
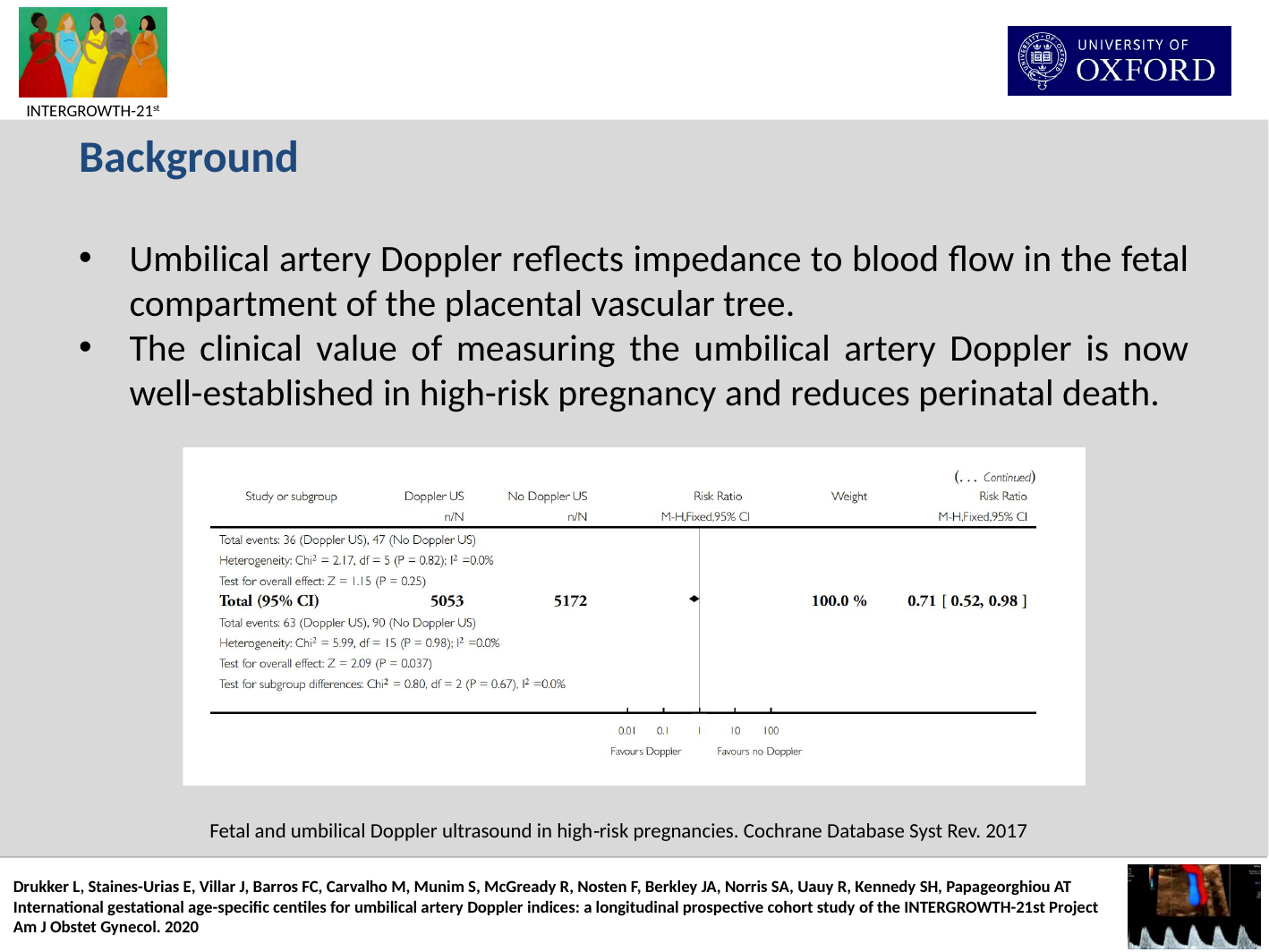

Background
Umbilical artery Doppler reflects impedance to blood flow in the fetal compartment of the placental vascular tree.
The clinical value of measuring the umbilical artery Doppler is now well-established in high-risk pregnancy and reduces perinatal death.
Fetal and umbilical Doppler ultrasound in high‐risk pregnancies. Cochrane Database Syst Rev. 2017

## Slide 3
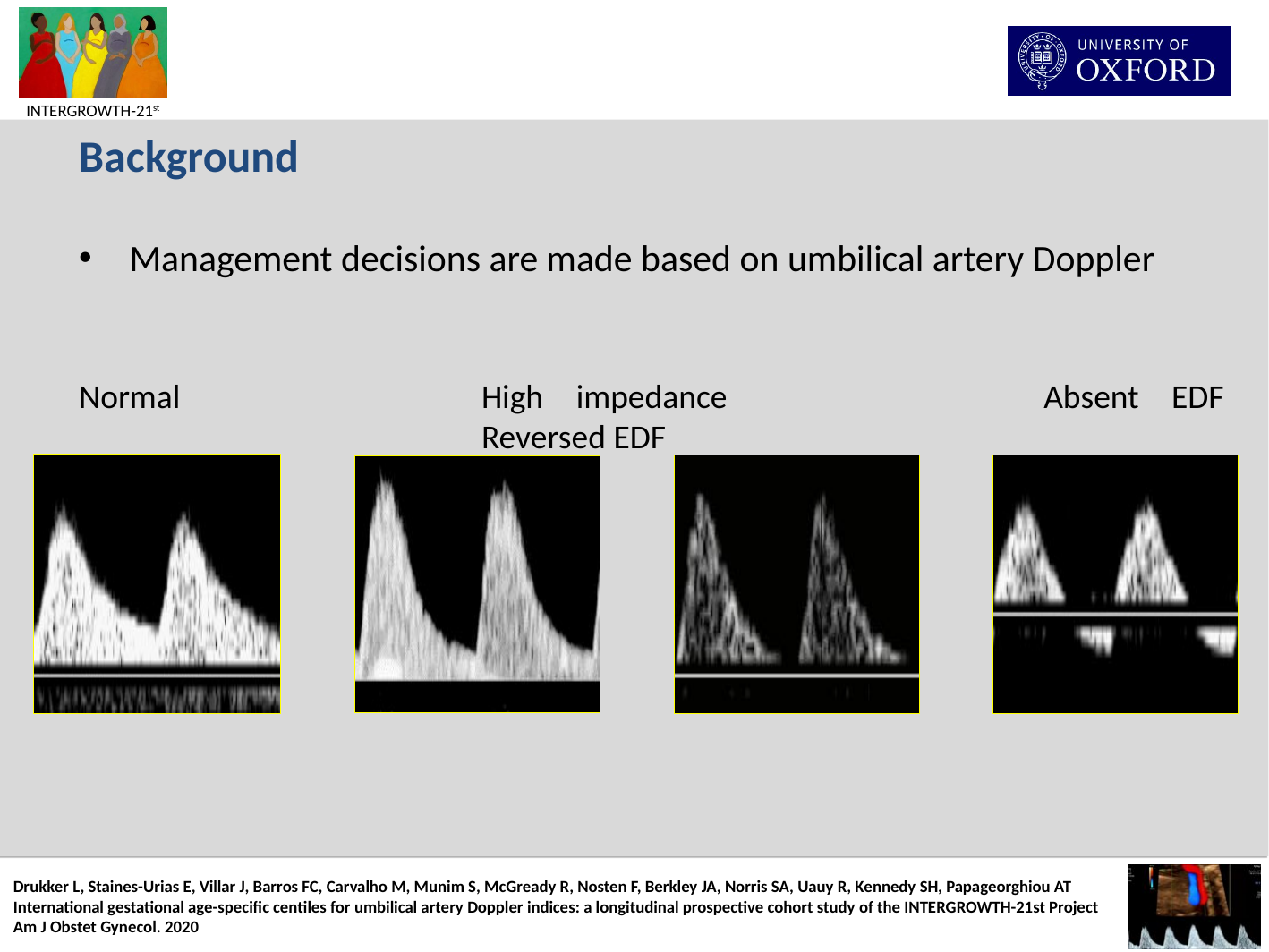

Background
Management decisions are made based on umbilical artery Doppler
Normal			High impedance			Absent EDF			Reversed EDF

## Slide 4
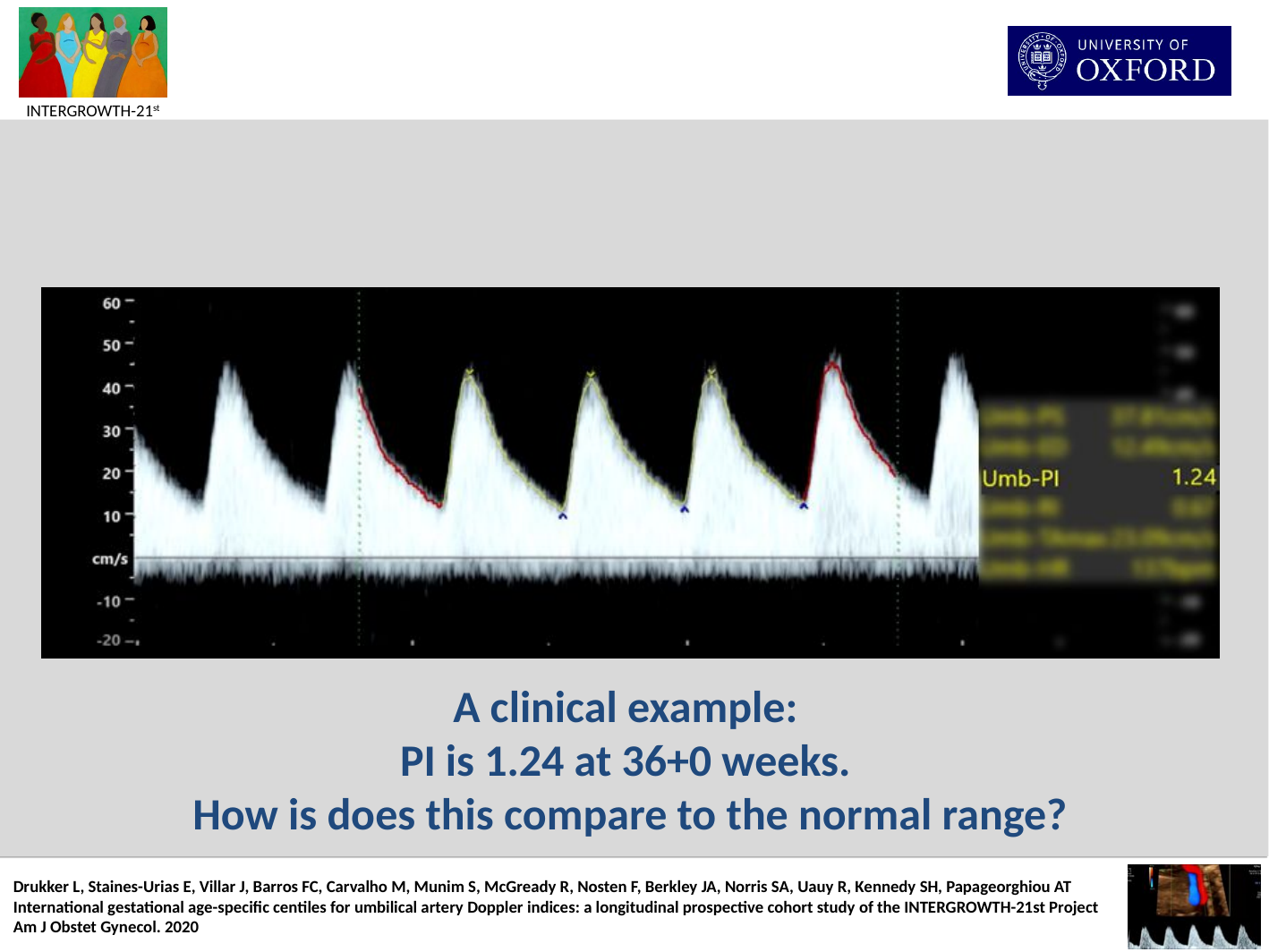

A clinical example:
PI is 1.24 at 36+0 weeks.
How is does this compare to the normal range?

## Slide 5
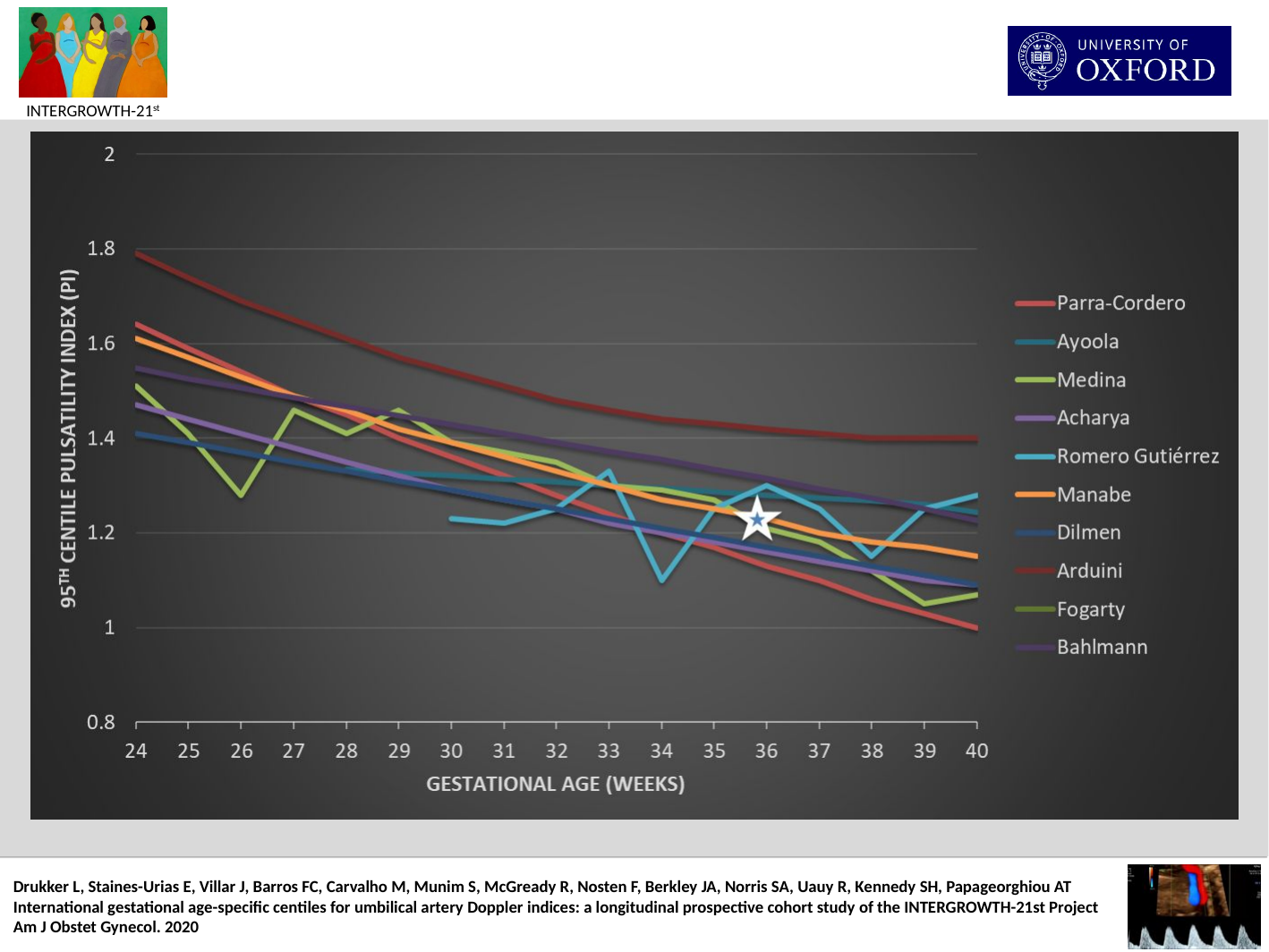

## Slide 6
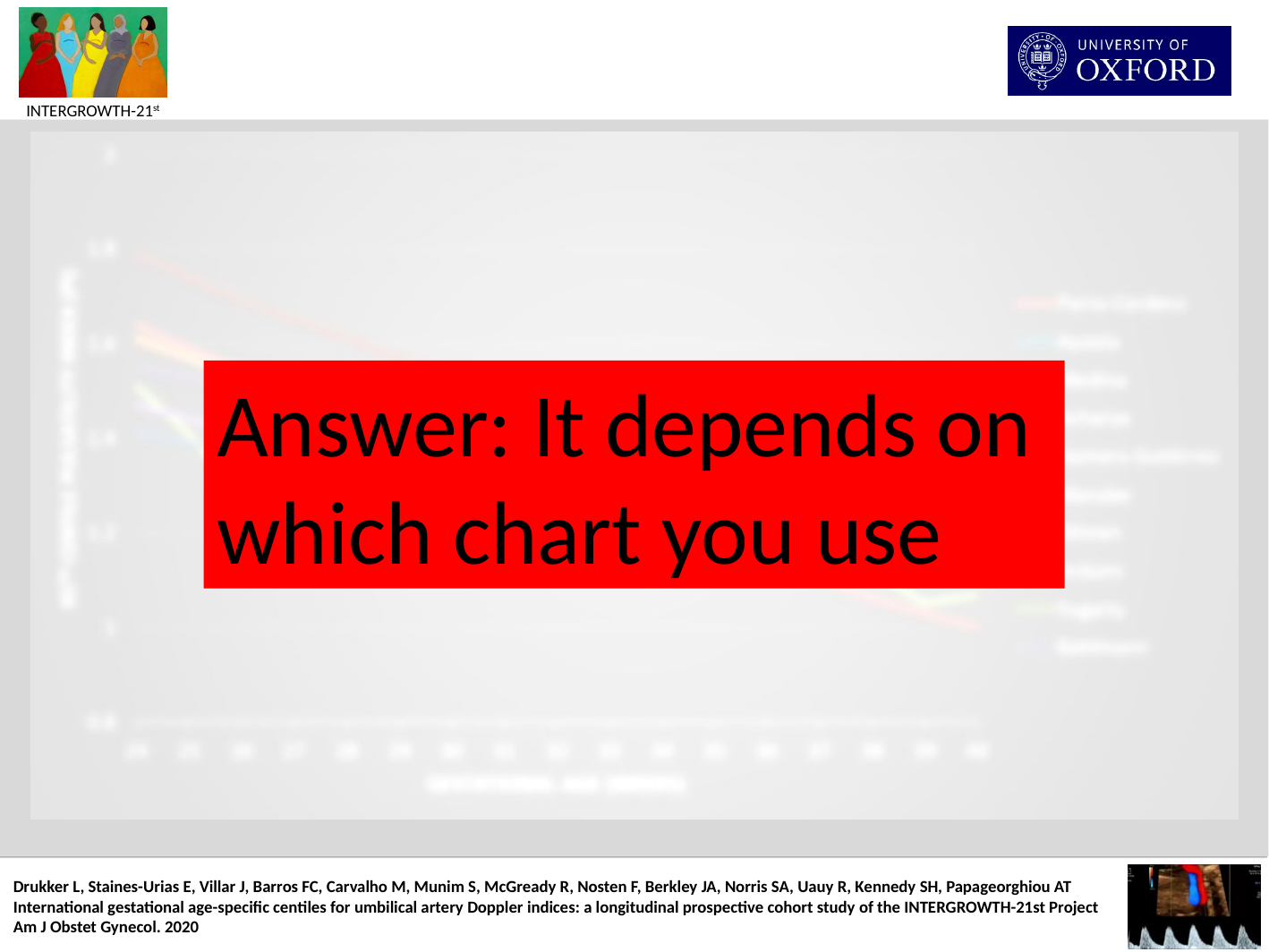

Answer: It depends on which chart you use

## Slide 7
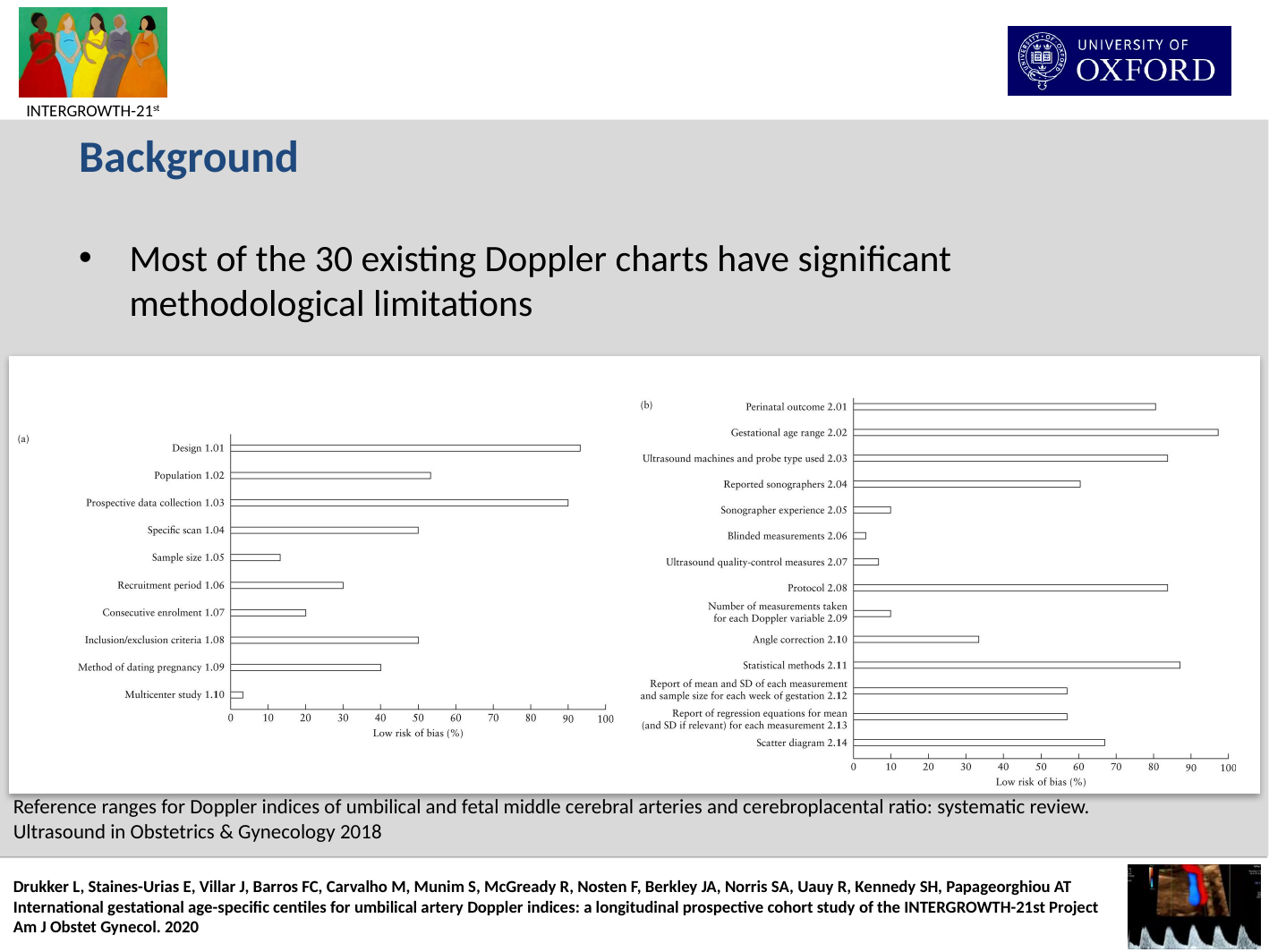

Background
Most of the 30 existing Doppler charts have significant methodological limitations
Reference ranges for Doppler indices of umbilical and fetal middle cerebral arteries and cerebroplacental ratio: systematic review. Ultrasound in Obstetrics & Gynecology 2018

## Slide 8
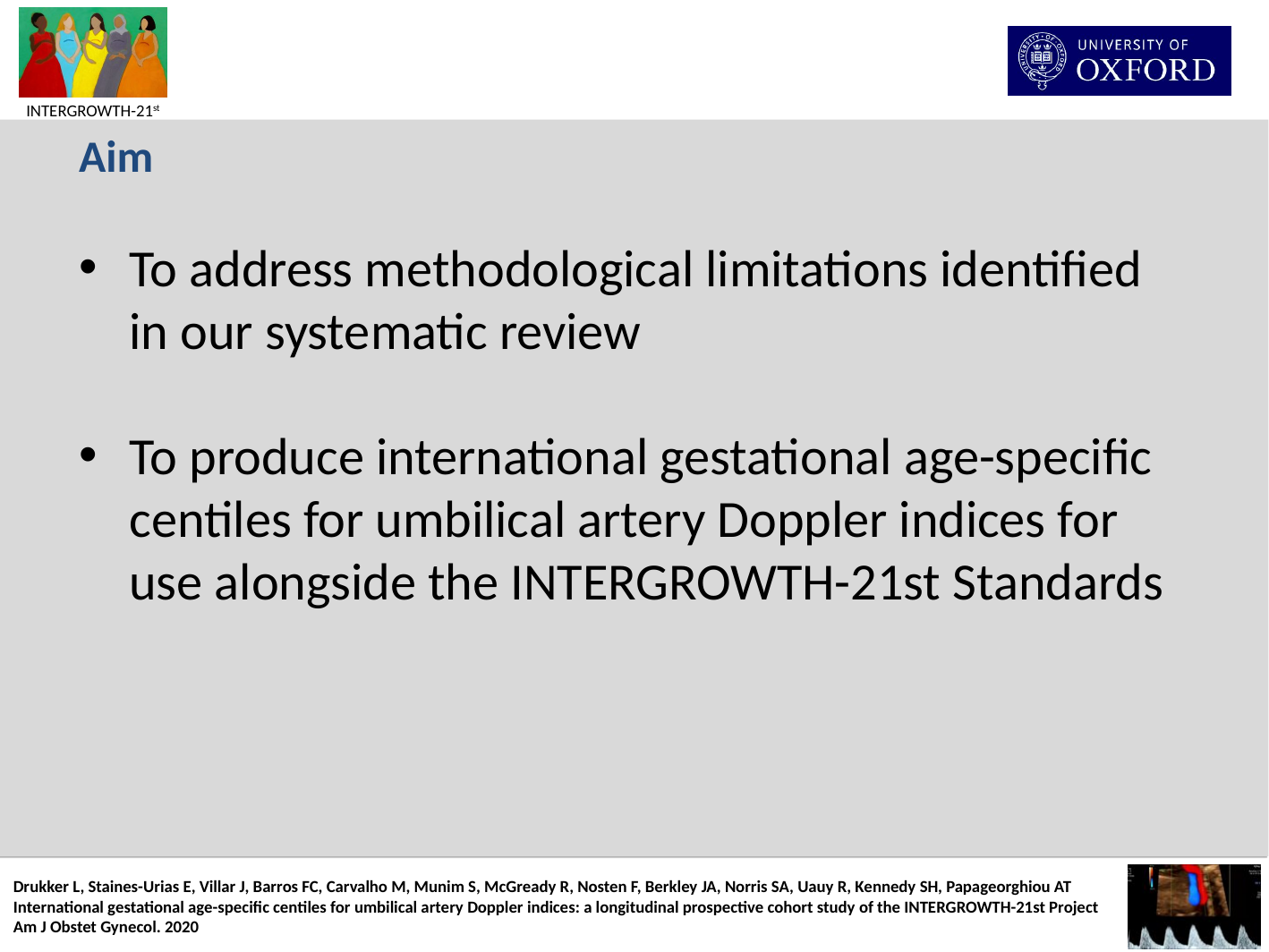

Aim
To address methodological limitations identified in our systematic review
To produce international gestational age-specific centiles for umbilical artery Doppler indices for use alongside the INTERGROWTH-21st Standards

## Slide 9
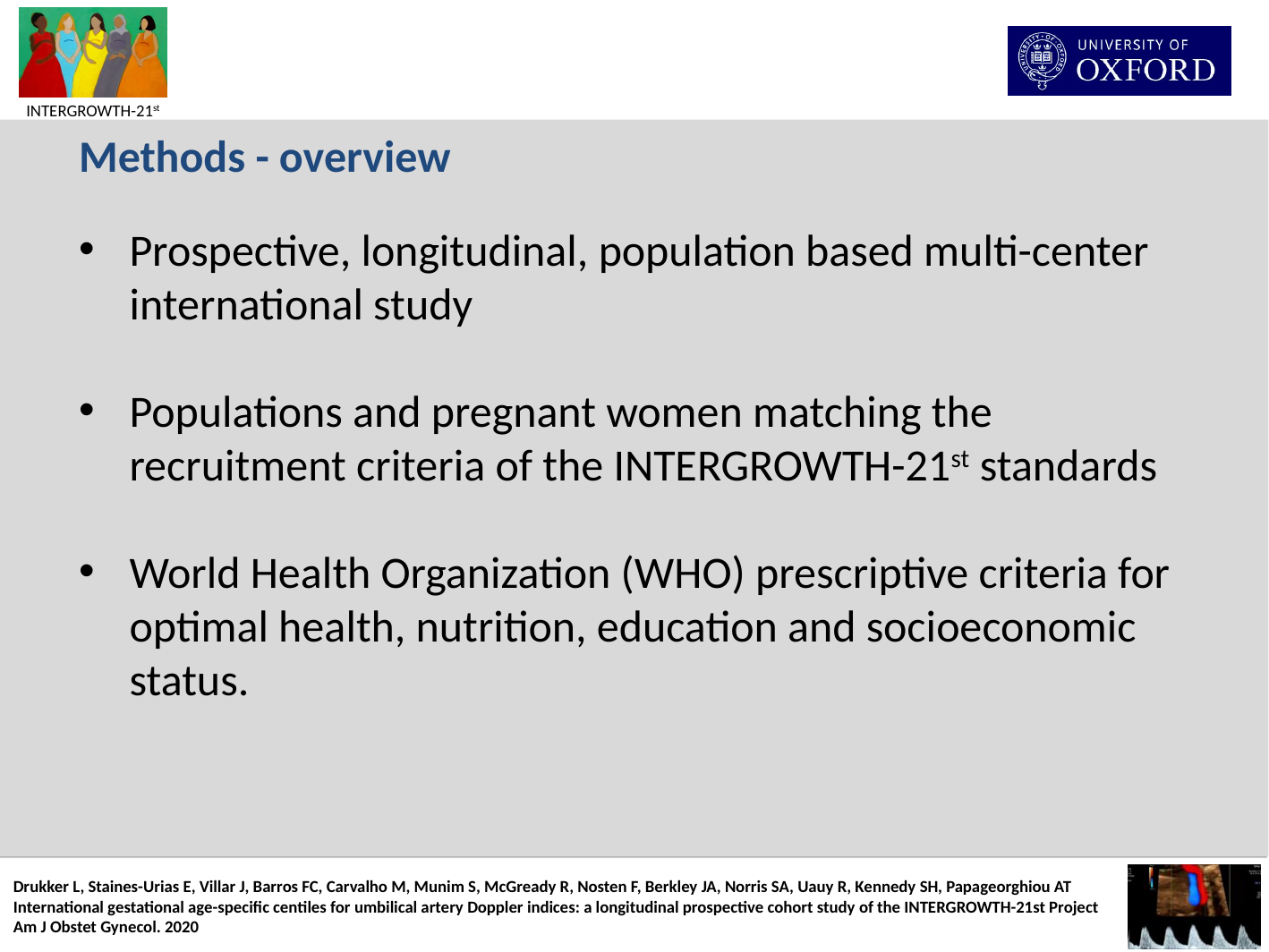

Methods - overview
Prospective, longitudinal, population based multi-center international study
Populations and pregnant women matching the recruitment criteria of the INTERGROWTH-21st standards
World Health Organization (WHO) prescriptive criteria for optimal health, nutrition, education and socioeconomic status.

## Slide 10
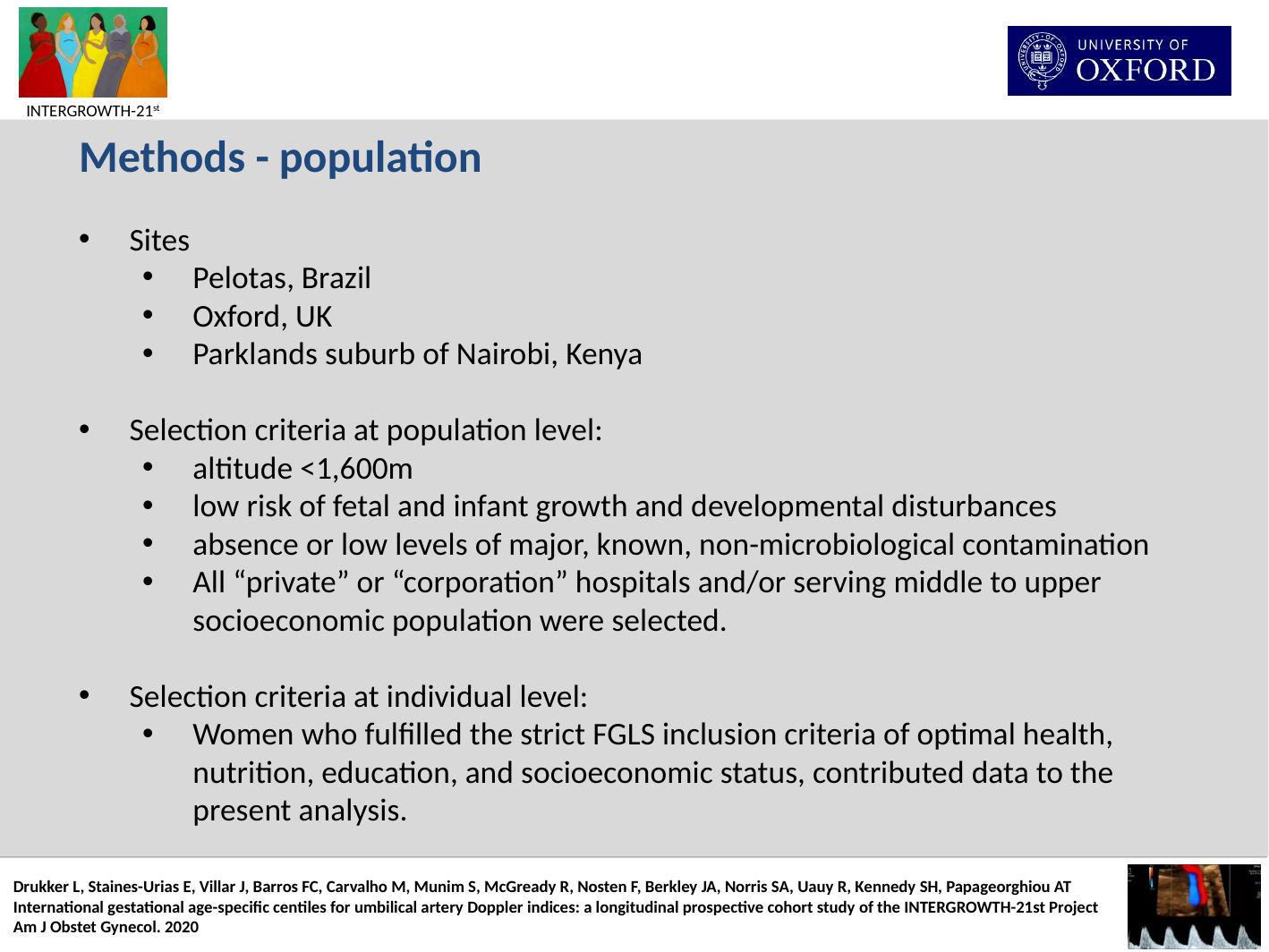

Methods - population
Sites
Pelotas, Brazil
Oxford, UK
Parklands suburb of Nairobi, Kenya
Selection criteria at population level:
altitude <1,600m
low risk of fetal and infant growth and developmental disturbances
absence or low levels of major, known, non-microbiological contamination
All “private” or “corporation” hospitals and/or serving middle to upper socioeconomic population were selected.
Selection criteria at individual level:
Women who fulfilled the strict FGLS inclusion criteria of optimal health, nutrition, education, and socioeconomic status, contributed data to the present analysis.

## Slide 11
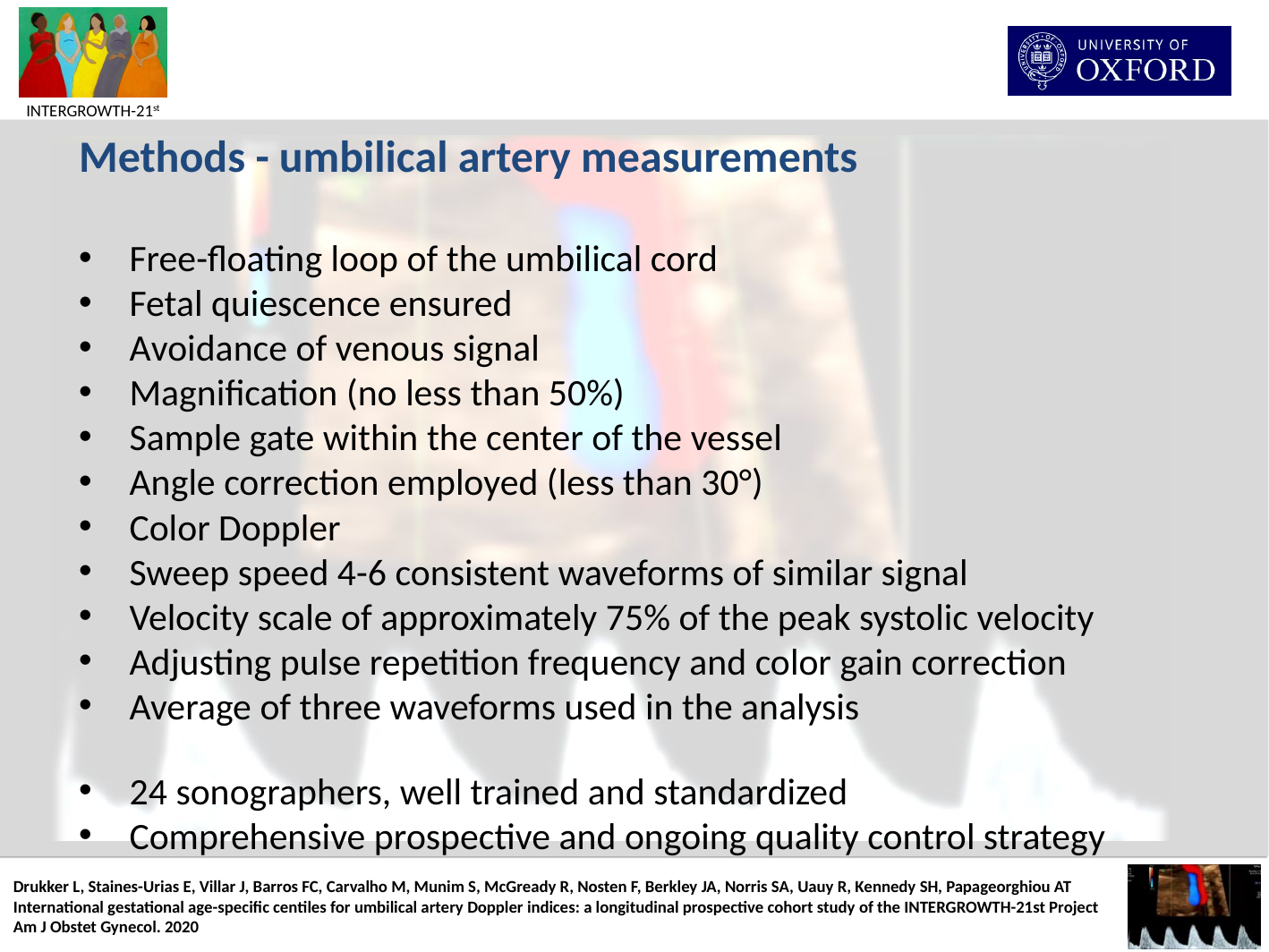

Methods - umbilical artery measurements
Free-floating loop of the umbilical cord
Fetal quiescence ensured
Avoidance of venous signal
Magnification (no less than 50%)
Sample gate within the center of the vessel
Angle correction employed (less than 30°)
Color Doppler
Sweep speed 4-6 consistent waveforms of similar signal
Velocity scale of approximately 75% of the peak systolic velocity
Adjusting pulse repetition frequency and color gain correction
Average of three waveforms used in the analysis
24 sonographers, well trained and standardized
Comprehensive prospective and ongoing quality control strategy

## Slide 12
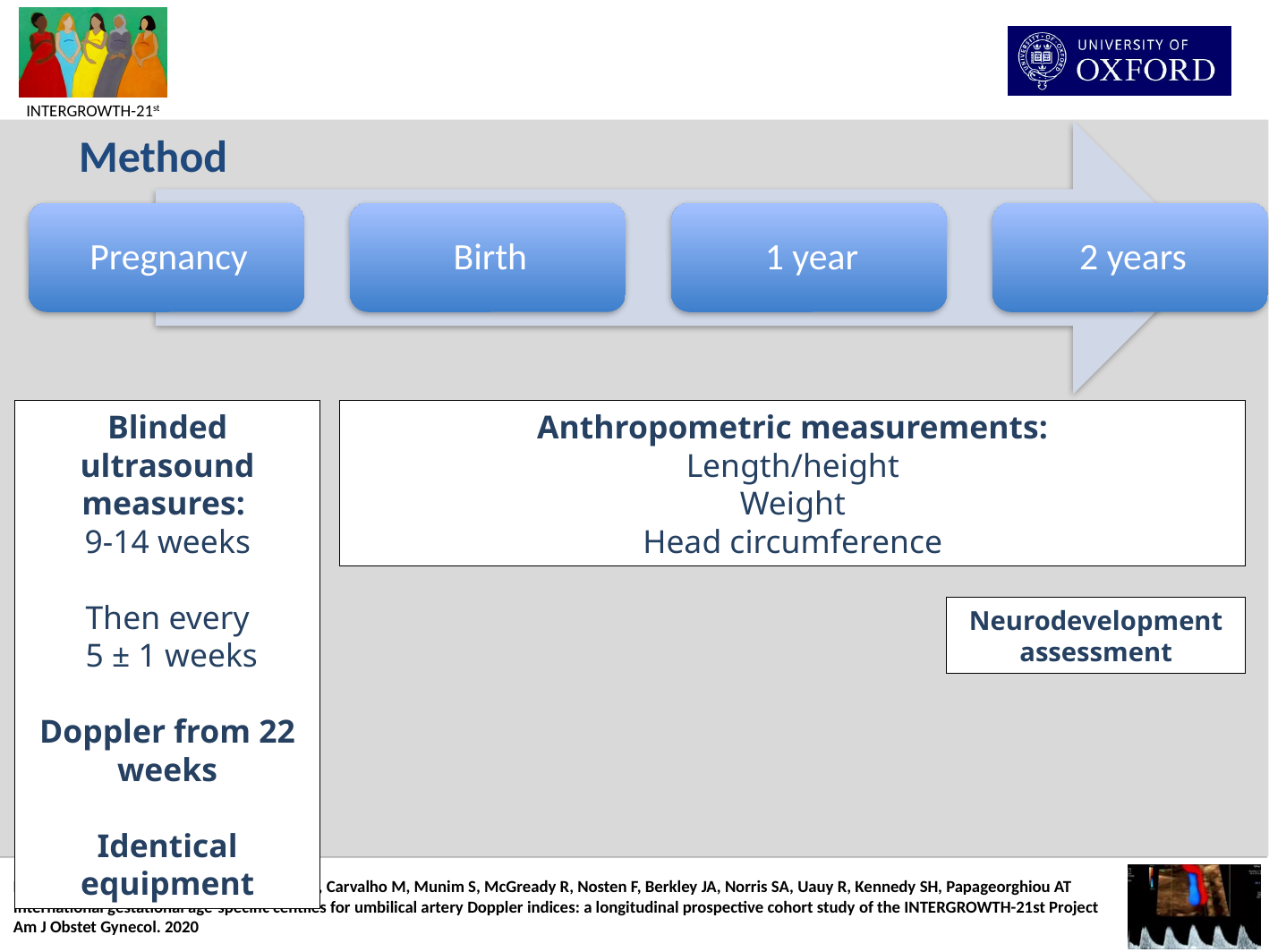

Method
Blinded ultrasound measures:
9-14 weeks
Then every
 5 ± 1 weeks
Doppler from 22 weeks
Identical equipment
Anthropometric measurements:
Length/height
Weight
Head circumference
Neurodevelopment assessment

## Slide 13
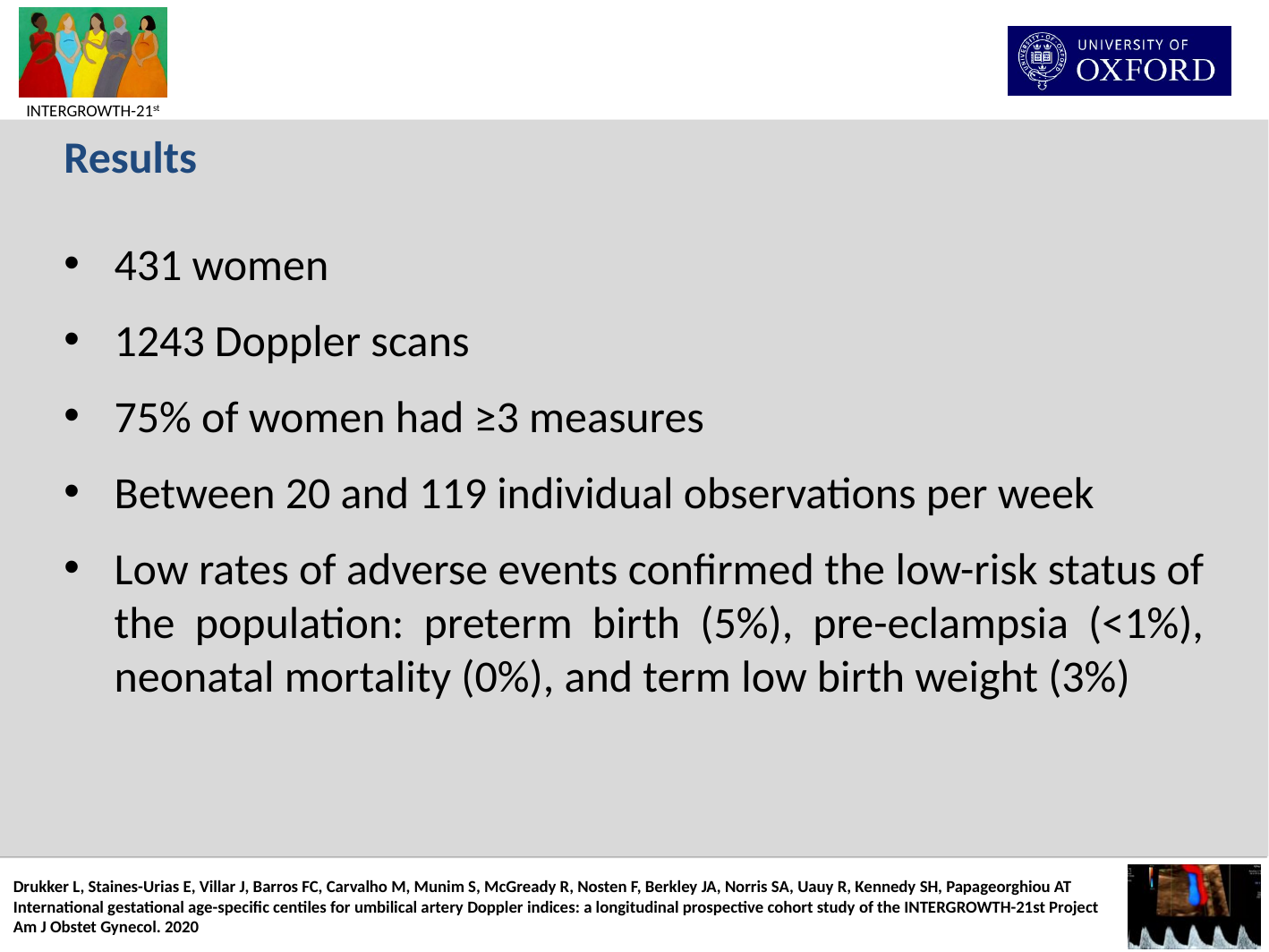

Results
431 women
1243 Doppler scans
75% of women had ≥3 measures
Between 20 and 119 individual observations per week
Low rates of adverse events confirmed the low-risk status of the population: preterm birth (5%), pre-eclampsia (<1%), neonatal mortality (0%), and term low birth weight (3%)

## Slide 14
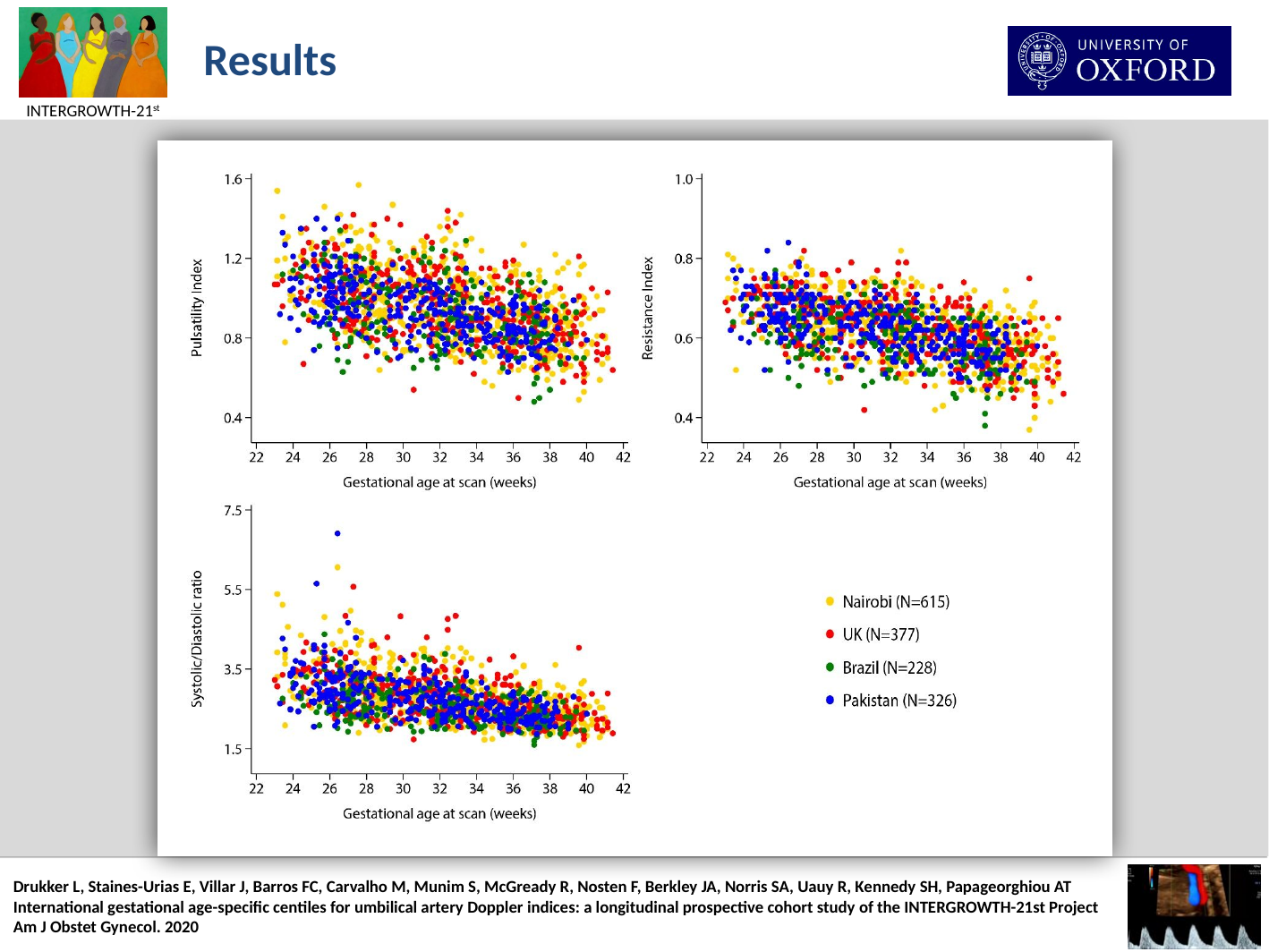

Results

## Slide 15
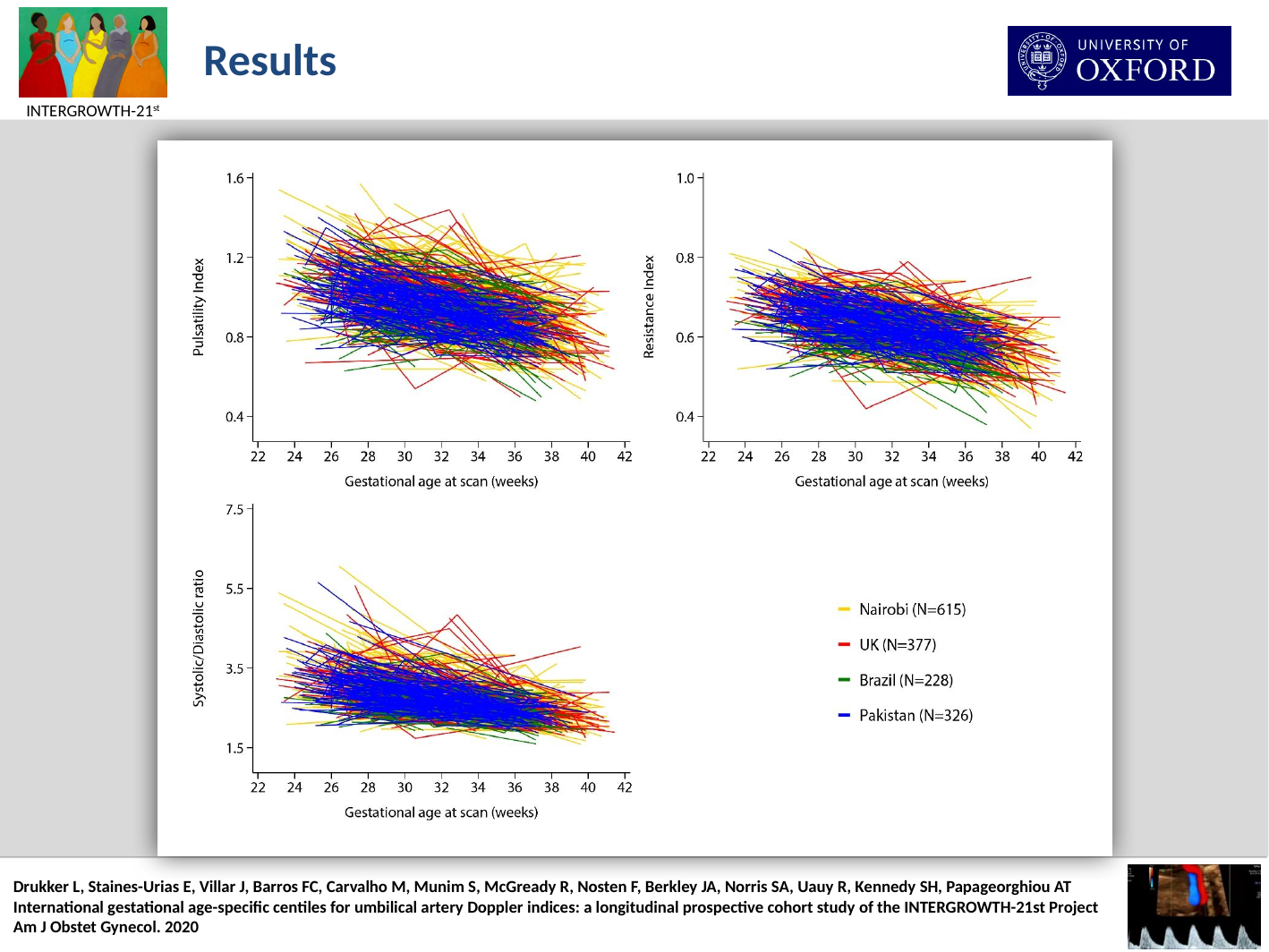

Results

## Slide 16
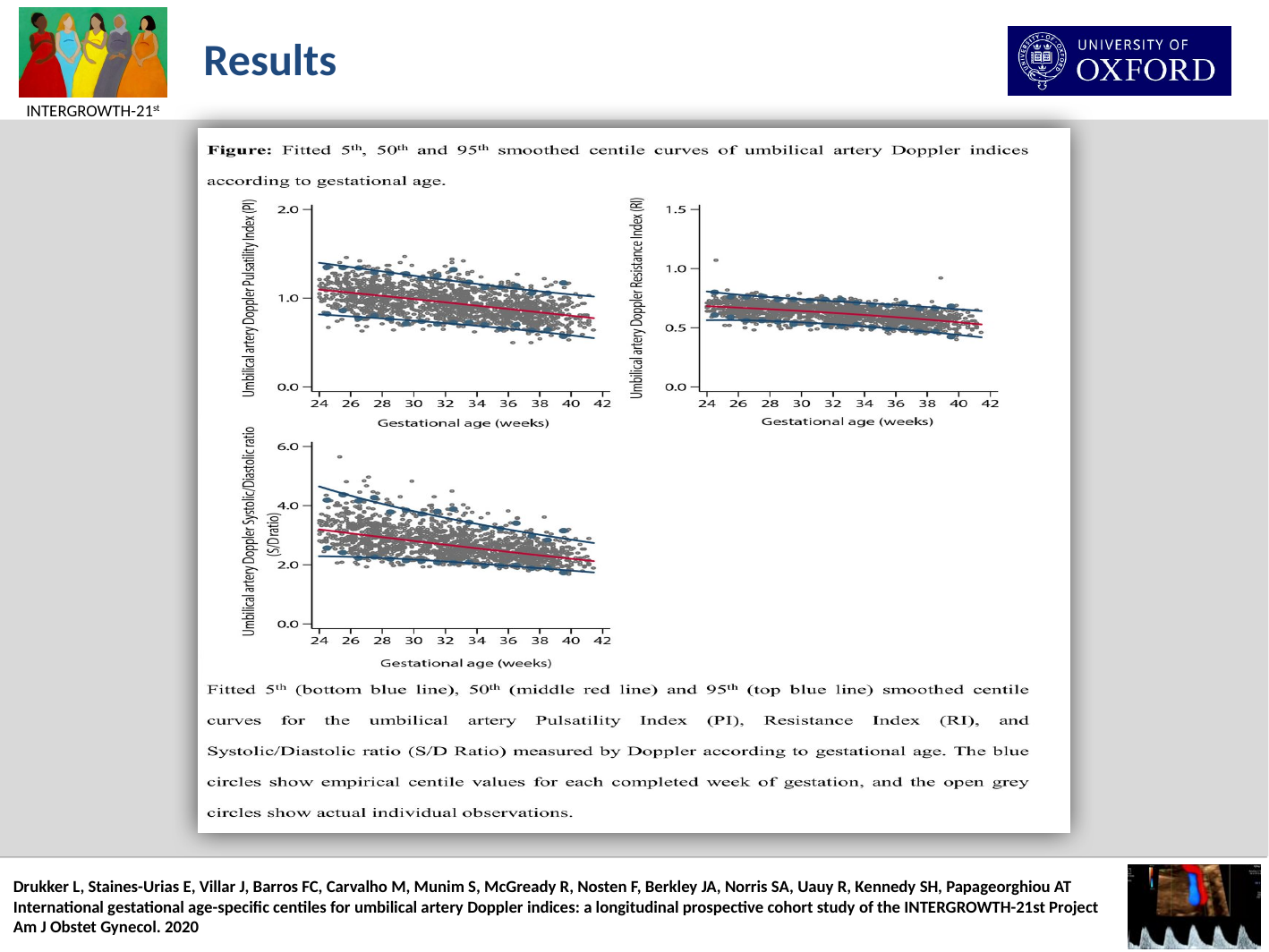

Results

## Slide 17
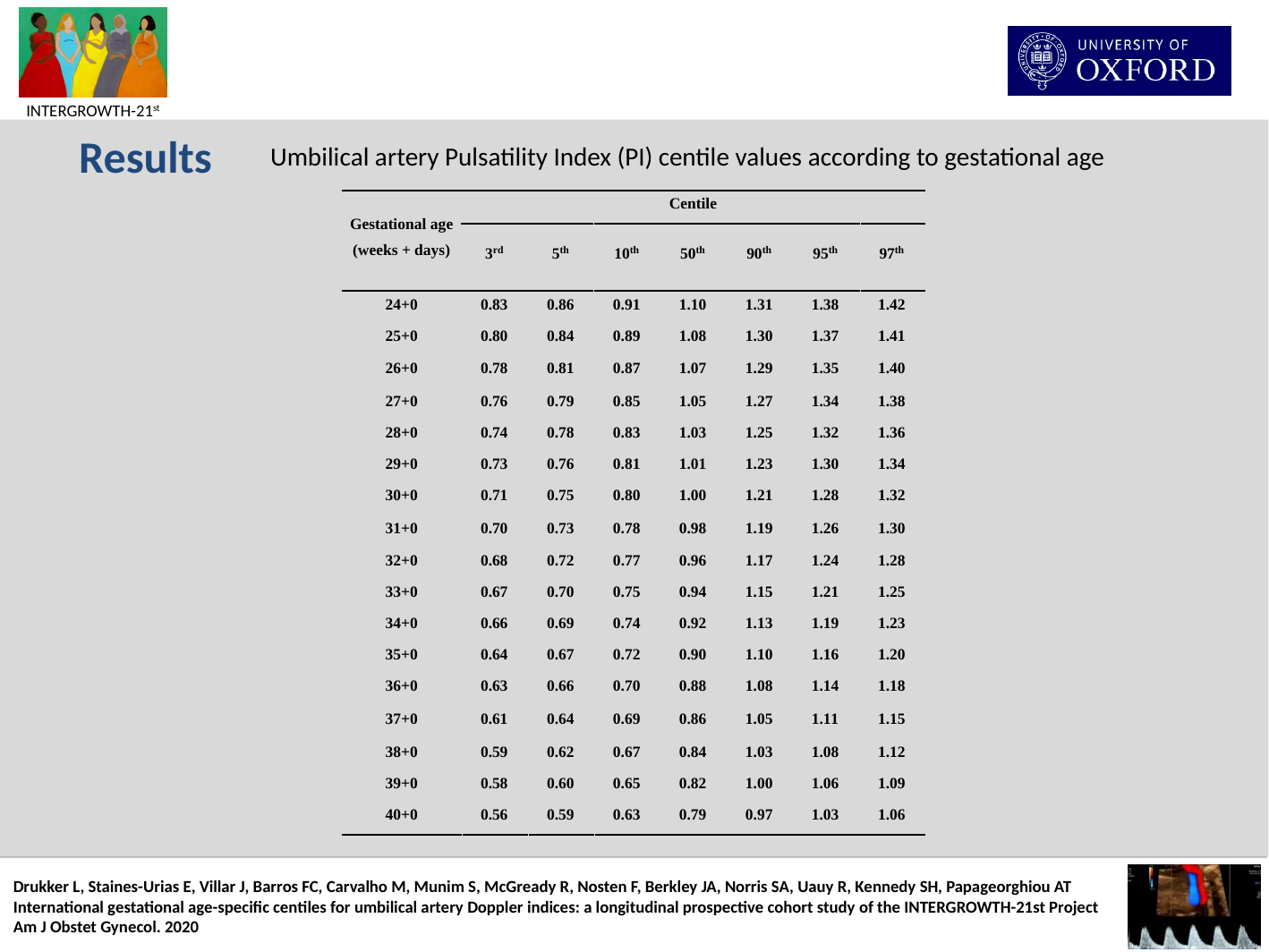

Results
Umbilical artery Pulsatility Index (PI) centile values according to gestational age

## Slide 18
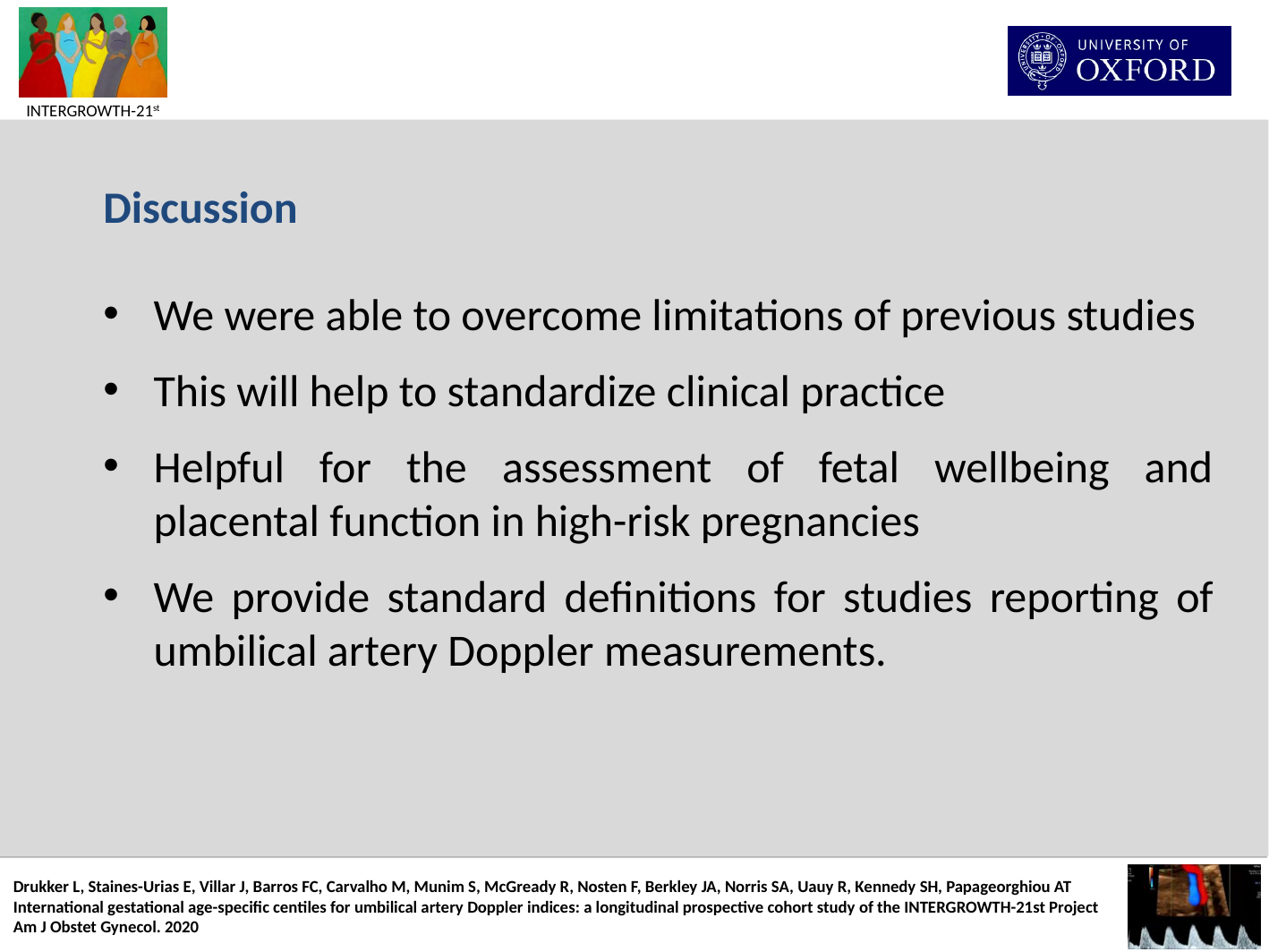

Discussion
We were able to overcome limitations of previous studies
This will help to standardize clinical practice
Helpful for the assessment of fetal wellbeing and placental function in high-risk pregnancies
We provide standard definitions for studies reporting of umbilical artery Doppler measurements.
